# Improving risk analysis of environmentally driven zoonotic biological threats as a primary pandemic prevention approach: A case study of the Tripartite Joint Risk Assessment Operational Tool operationalization in Kenya

**DOI:** 10.1371/journal.pgph.0006560

**Published:** 2026-07-01

**Authors:** Oluwayemisi Ajumobi, Anthony Etyang, Kenneth Munge Kabubei, Kadondi Kasera, Rabera Kenyanya, Meghan Davis, Christine Marie George, Sophie von DobSchuetz, Crystal Watson, Jennifer Nuzzo

**Affiliations:** 1 Department of Environmental Health and Egineeering, Johns Hopkins University Bloomberg School of Public Health, Baltimore, Maryland, United States of America; 2 Kenya Medical Research Institute (KEMRI) KEMRI- Wellcome Trust Research Programme, Nairobi, Kenya; 3 World Bank Group, Nairobi, Kenya; 4 Kenya Ministry of Health, Nairobi, Kenya; 5 Kenya Biovax Institute, Nairobi, Kenya; 6 World Health Organization, Geneva, Switzerland; 7 Department of Epidemiology, Brown University School of Public Health, Providence, Rhode Island, United States of America; Institute of Development Studies, UNITED KINGDOM OF GREAT BRITAIN AND NORTHERN IRELAND

## Abstract

Kenya faces a heightened risk of emerging and/or reemerging infectious disease (EID) outbreaks of zoonotic origins due to climate-induced extreme weather events and other environmental drivers. We aimed to obtain the perspectives of stakeholders on the development of a newly conceptualized integrated risk analysis (IRA) framework to address environmentally driven EIDs. We addressed the study aim by learning from stakeholders’ experiences with the operationalization of the Joint Risk Assessment Operational Tool (JRA OT) in Kenya. We conducted an exploratory and explanatory qualitative study using a constructivist approach. National- and county-level government representatives, representatives from the Quadripartite institutions (FAO/UNEP/WHO/WOAH), and other partner institutions related to One Health (OH) in Kenya were recruited to participate in semi-structured key informant interviews from 22/02/2024 to 30/03/2024. A total of twenty-eight interviews were conducted, transcribed verbatim and analyzed using a thematic framework analysis. Study participant pool comprised sixteen, five, and seven county-level, national-level, and partner institution representatives respectively. Four major themes emerged from the study data as essential components of an IRA framework: (i) Improving cross-sectoral data sharing and integration; (ii) Early stakeholder inclusion and holistic engagement; (iii) Proactive cross-sectoral resource mobilization and allocation; and (iv) Policies and legislation for addressing OH governance challenges. Improving IRA processes to ensure integration of the environment sector into the OH approach will enhance preparedness for and response to environmentally driven zoonotic biological threats. National and sub-national OH policies are needed to enable routine operationalization of risk-based OH activities like Joint Risk Assessments (JRAs) and other aspects of risk analysis to inform cross-sectoral decision making for pandemic prevention.

## Introduction

Kenya is a climate-vulnerable country in East Africa that is prone to extreme weather events (EWEs) such as severe droughts and flooding. Increasing frequency and severity of climate-induced EWEs in Kenya are resulting in increased risk of infectious disease outbreaks and significant socioeconomic and environmental impact [1]. Recently, the November 2023 El Nino and the April-May 2024 positive Indian Ocean Dipole climatic patterns in the country intensified the short and long rainy seasons that typically occur annually from October to December and from Mid-March to May respectively [2,3]. These and other past climatic conditions have resulted in severe flooding events across the country and associated outbreaks of infectious diseases [2,4].

Across the East Africa region, environmental drivers such as climate change, habitat encroachment, and land use change are expanding the range of emerging and/or reemerging infectious disease (EID) pathogens to non-endemic countries in the region including Kenya [5–7]. Hence, in addition to priority endemic zoonotic diseases (such as anthrax, brucellosis, bovine tuberculosis, and rabies) listed under the 2021–2025 National One Health Strategic Plan [8]. EIDs are also a major concern to the Government of Kenya and are prioritized in the strategic plan. EID pathogens of focus in this study include arthropod transmissible pathogens of zoonotic origins like Crimean-Congo haemorrhagic fever virus (CCHFV) and non-arthropod transmissible EID pathogens like Marburg virus (MARV) and Middle East respiratory syndrome coronavirus (MERS-CoV). The rationale for the selection of these three pathogens include increasing evidence of CCHFV and MERS-CoV seroprevalence across Kenya linked to climate change-driven changes in animal, human and wildlife population migration patterns and risk of environmentally driven MARV spillover events and potential to sustain human-to-human disease transmission [9–12].

Previous studies have called for a better understanding and synthesis of probable environmentally driven disease transmission risk pathways as part of integrated risk assessments to improve risk characterization and to inform prevention, preparedness, and response strategies [13–15]. A study by Loh et al. (2015) explored the major environmental drivers of EIDs of zoonotic origins and highlighted the importance of targeting surveillance and control activities to address different transmission pathways [13]. To emphasize the need for a better understanding of the eco-social processes that facilitate disease emergence and spread under risk analysis, Jones et al (2016) provided examples of emergence and spread of different pathogens including previously unknown pathogens like Nipah virus, MERS-CoV, and Avian Influenza; and pathogens with extended geographic range like West Nile virus, and Ebola virus [14]. Similarly, Sorvillo et al. (2020) discussed the need for joint risk assessments of zoonotic biological threats like CCHFV by utilizing a comprehensive framework that combines epidemiological, ecological, virology and vector biology data, with modeling data to make an estimation of outbreak risk and target surveillance activities [15].

Consolidated assessment tools such as the Joint Risk Assessment Operational Tool (JRA OT) of the former Tripartite [comprised Food and Agriculture Organization (FAO), World Health Organization (WHO), and World Organisation for Animal Health (WOAH)] are utilized by national ministries across relevant sectors (including the animal, human, environment, and wildlife sectors) and other technical partners for zoonotic biothreats risk assessments using a transdisciplinary One Health (OH) approach. The JRA OT is one of three operational tools developed in 2020 under the Tripartite Zoonosis guide to support countries like Kenya with the application of a consistent and harmonized cross-sectoral approach to qualitatively assess risks posed by zoonotic disease hazards at the human-animal-environment interfaces, and at the national and sub-national levels [16]. However, there remain gaps in accounting for environmental drivers in existing risk assessment tools and overall risk analysis processes in relation to pathogen/disease spillover, emergence and spread [17]. These gaps include the need for expanding the application of existing tools to biological threats related to environmental hazards; and enhancing the integration of the United Nations Environment Programme (UNEP), the fourth member of the recently expanded Quadripartite institutions (inclusive of FAO, WHO, and WOAH) and the environment sector in the operationalization of cross-sectoral risk-based OH processes. Within the study context, in line with the UNEP Strategy for Environmental Education and Training, the environment sector refers to industries and activities encompassing the natural and built environment, sociological and economic aspects of environmental issues, and the political dimensions of environmental protection and resource management [18]. Further, as classified under the Index for Risk Management Global Risk Index (INFORM GRI), environmental hazards include floods, droughts, earthquakes, tsunamis, tropical cyclones, and epidemics [19].

This study aimed to obtain the perspectives of stakeholders on the development of a newly conceptualized integrated framework for risk analyses of environmentally driven EIDs [with a focus on Crimean-Congo haemorrhagic fever (CCHF), Marburg virus disease (MVD), and Middle East respiratory syndrome (MERS)] to inform cross-sectoral decision-making for pandemic prevention using a OH approach [20]. We addressed the study aim by conducting key informant (KI) interviews to learn from the experiences of stakeholders with the operationalization of the JRA OT in Kenya and to obtain their inputs on essential components required to improve environmentally driven biological threat risk analysis processes at the environment-animal-human exposure interface. Information obtained from the study was used to finalize the development of an integrated risk analysis (IRA) framework for pathogen/disease spillover, emergence/reemergence and spread (SES) risk analysis to inform cross-sectoral pandemic prevention decision making.

## Methods

### Study design and participants

The single qualitative case study was carried out in Kenya as an exploratory and explanatory study that utilized a constructivist approach [21]. A qualitative study design was adopted based on the need to learn from and capture the unique perspectives of KIs, including those responsible for operationalizing OH at the national- and sub-national (county)- levels, on the IRA framework development. In addition to the increasing vulnerabilities to EIDs and climate change induced EWEs, Kenya was selected as the country setting to reflect the country’s status as one of the first countries to operationalize and institutionalize the JRA OT for the cross-sectoral joint risk assessment (JRA) of zoonotic biological threats at national- and county- levels to improve national preparedness and response planning efforts.

The study involved semi-structured KI interviews with national- and county-level governments, members of the Quadripartite institutions (FAO/UNEP/WHO/WOAH) and other partner organizations from the animal, human, environment, and wildlife sectors. Participants were eligible to participate in the study if they were familiar and/or had experience with the use of the JRA OT for the assessment of at least two prior zoonotic biological threats; represented a sector under the Kenya National OH platform, also called the Zoonotic Disease Unit (ZDU) and serves as the JRA Steering Committee; and expressed willingness to participate in the study. All eligibility criteria were required to participate in the study

Potential participants were identified and nominated by the ZDU from a list of attendees of past JRA OT training workshops and other related OH trainings. We utilized a stratified purposeful and maximum variation sampling technique for the nomination of participants from a larger list to ensure ample diversity in representation of participants at different level of engagements (national- and county-level governments, and partner institutions) and across different counties and sectors. Snowballing was also used to identify additional participants. Selection of counties was conducted via a purposeful sampling strategy. This strategy accounted for the high-risk profiles of counties selected by the ZDU as the first cohort of counties trained in the utilization of the JRA OT for the assessment of zoonotic biological threats. These counties include Bungoma, Busia, Kakamega, Marsabit, and Vihiga. Participants from three additional counties (Bomet, Kitui, and Tana River) were selected based on the increasing risk profiles of the counties and the support provided to the national government with the operationalization of the JRA process.

Participants were invited to participate in the study via email sent to participants between February to March 2024. In addition to the primary email nomination invites sent out by the ZDU, five potential participants from the Quadripartite were identified by their colleagues via snowball sampling based on their earlier engagement/familiarity with the JRA process and their areas of sectoral expertise. These five participants were also sent email nomination invites to participate in the study. For the final study sample, one participant from a partner institution declined, three participants representing both a partner institution and government agency were unavailable due to scheduling conflicts (n = 2) and a transfer to a different county (n = 1), and all other participants agreed to participate in the study. The study team followed the consolidated criteria for reporting qualitative research (COREQ) checklist guidelines for reporting the study information provided in this manuscript [22].

### Ethics statement

The study was approved by the African Medical and Research Foundation (AMREF) Ethics and Scientific Review Committee (approval number ESRC P1550/2023) and the Kenya National Commission for Science Technology and Innovation. Johns Hopkins University Institutional Review Board granted the study a non-human subject research exemption. Participants also gave their oral informed consent to participate in the study prior to the interviews. Each participant’s oral informed consent was documented using an oral informed consent form that was read out to participants prior to the start of the interviews. The ESRC approved the use of oral consent as a more appropriate form of obtaining participant consent to participate in the study to address sensitivities with collecting identifying information.

### Data collection procedure

For data collection, we developed an interview guide using key elements of the initially developed IRA conceptual framework described in a related study by Ajumobi et al (2025) [20]. This framework was adapted from INFORM GRI framework that utilizes a multihazards approach to improve the evidence base for epidemic risk analysis [19]; and the WHO Health Emergency and Disaster Risk Management (Health EDRM) Framework for assessing, mitigating, and communicating risks across different inflection points and along the continuum of prevention and risk management [23]. We conducted a total of 28 semi-structured 1:1 interviews (45–60 minutes long) in English (20 in-person; 8 virtual) during the study recruitment period from 22/02/2024 to 30/03/2024. The team had anticipated conducting no more than 30 interviews to achieve data saturation; data collection was completed after reaching theoretical saturation.

The twenty in-person interviews were primarily carried out with KIs representing national- and county-level governments, and partner organizations; and included visits to five counties (Bungoma, Busia, Kakamega, Vihiga, and Nakuru) and Nairobi as study sites. The in-person interviews were scheduled with participants at an agreed convenient location. Most of the in-person interviews took place at the work location of participants, while two interviews were conducted at a public café.

In addition to the in-person interviews, four virtual interviews were conducted with KIs from four counties (Bomet, Kitui, Marsabit, and Tana River). An additional four virtual interviews were scheduled with KIs who were unavailable during the in-person visits and requested a virtual option as an alternative arrangement. All interviews were recorded using a handheld audio recording device. Field notes were also taken by a research assistant who participated in the county visits and served as a second coder on the research team.

Following the completion of the interviews, a stakeholder validation workshop was conducted with a sub-group of study participants (n = 14) purposively selected to serve as a representative sample of the total participant pool. Study findings were presented at the validation workshop and interactive group sessions were held with study participants to validate components of the IRA framework and other findings that emerged from the study data. Meeting notes were taken at the workshop and the main workshop sessions were recorded using the audio recording device used for the interviews.

The KI interview guide utilized for data collection is attached as a supporting information (S1 Text).

### Data analysis

Interview audio files were transcribed both manually and using an institutionally approved transcription service (Qualtranscribe; Boston, MA). We used NVivo 14 (Lumivero; Denver, CO) to upload the interview transcripts for coding, data management and analysis. A thematic framework analysis was utilized for data analysis informed by the components of the study’s conceptual framework and used to develop a codebook. This process entailed using an embedded technique showing correspondence of the study objective with the conceptual framework and utilizing components of the framework to guide the data analysis. The thematic framework analysis template used for the analysis is attached as a supporting information (S1 Table). Reflexive notes were also documented throughout the data collection and analysis process (*Panel 1*).

**Panel 1: Reflexivity.**

The study team recognized the self-identity of the primary investigator as an African woman working in a male-dominated field. To reduce assumptions about how researcher identity and preconceived notions of participants may influence the quality of collected data, it was important to acknowledge the role that the self-identity of the investigator may play in the process. Additionally, the investigator’s prior experience working on OH issues across different countries in Sub-Saharan Africa has highlighted gaps in coordination, collaboration, and coordination of the animal and human health sector with the environment sector. These gaps are viewed as a primary challenge hindering the implementation of the OH approach in the region. Hence the study team considered underlying assumptions about existing gaps in the risk pathway analysis due to preconceived OH challenges. To reduce the influence of these assumptions, KIs were provided ample opportunities to share context-specific examples of OH operationalization challenges in Kenya using open-ended questions. Where appropriate, participants were also encouraged to provide perspectives on how these challenges are comparable with challenges experienced in other country settings. Overall, these strategies served to reduce both participant and researcher bias.

Before commencing the coding process, the codebook containing the main codes and sub-codes were extensively discussed between the two coders including the descriptions for each code. Preliminary data analysis was carried out concurrently with data collection and involved the use of a deductive coding strategy whereby main codes and sub-codes were pre-identified from the framework and interview guide, in line with the study objectives. An inductive coding strategy was also used to identify and add new codes emerging from the data to the codebook and the newly added codes were discussed with the second coder. By the 25^th^ interview, there was ample evidence of thematic saturation.

At the end of data collection, full data analysis involved re-reading the transcripts several times for better clarity of the main themes and sub-themes emerging from the data. To ensure reliability of the coding process, intercoder checks were conducted by coding ten percent of the interview transcripts and carrying out a manual comparison of the coded data. The fully coded data were also reviewed using a framework matrix to ensure the data were appropriately coded. At the end of coding, coded texts were discussed and categorized under categories pre-identified from the framework and using new categories identified during coding. Emerging themes and sub-themes were revealed after a careful study of the data grouped by categories and codes. An audit trail was used to keep track of the steps taken from data collection to data analysis.

## Results

### Sample description

KIs from the county government represented the highest number of participants (n = 16; 57%). Participants were from the animal/veterinary health, environment, human health, and wildlife; and worked on different areas of specialization including environmental sciences, natural resources and climate change, OH coordination, disease surveillance and response, infectious diseases research, and resource mobilization (Table 1). Overall, a majority of the 28 study participants were male (n = 23; 82%).

**Table 1:** Demographic data of study participants.

Demographic Variable	Participants (n = 28)
Institutional Level of Representation	
County	16 (57.0%)
Partners	7 (25.0%)
National	5 (18.0%)
Sectoral Level of Representation	
Human Health	10 (36.0%)
Animal/Veterinary Health	9 (32%)
Environment	7 (25%)
Wildlife	2 (7%)
Areas of Specialization	
One Health Coordination	7 (25.0%)
Veterinary Services	6 (22.0%)
Environmental Sciences, Natural Resources and Climate Change	4 (14.0%)
Public Health, Disease Surveillance and Response	4 (14.0%)
Infectious Disease Research (Human and Veterinary)	3 (11.0%)
Pharmacoepidemiology/Antimicrobial Resistance	1 (3.5%)
Environmental Policy and Regulation	1 (3.5%)
Ecology	1 (3.5%)
Resource Mobilization	1 (3.5%)
Gender	
Male	23 (82.0%)
Female	5 (18.0%)

### JRA OT operationalization: Best practices and lessons learned

Overall, there was consensus from most study participants about the importance and usefulness of the JRA OT training and operationalization in Kenya. KIs reported that the trainings helped introduce more proactive thinking to enable better preparedness for joint outbreak response and decision making. KIs also highlighted that a major outcome of the JRA was in facilitating OH operational planning of counties for the implementation of functional OH structures called County One Health Units (COHUs) and encouraging planning for country-wide scale up of COHUs across the forty-seven counties of Kenya.

*“We are actually now working on a veterinary services management bill that will be cascaded at the county level. We have a national one, but now at the county level, we are deliberately inserting some sections that will operationalize the COHU.”* – County D Participant

Lessons learned from the operationalization of the tool include more involvement and ownership of OH by the environment and other relevant sectors; the need to address gaps in OH operationalization, especially at the sub-national (county level) by implementing functional operational structures under the COHUs; and preference for more practice-oriented field-based JRAs.

*“.. for animal and human health, we have appointed people to handle issues during such emergencies that concern both sectors. But now, other sectors that are supposed to be locked in, like environment, we don’t have experts in those areas that we can be able to rely on when we are doing risk assessments.”* – County E Participant

*“Yeah, so I think what is required is that we have had One Health up there at the national level but little or nothing is happening at the sub national level...” -* County A Participant

*“The approach was good, but I think most of the participants in there wished for more emphasis on the practical aspect. If you are able to facilitate me doing that in the field, like going to the Busia border and assessing the risk of introduction of Avian Influenza or something like that. That one sticks more”* – Research Partner D2

**Panel 2: Thematic components and sub-components of the core Integrated Risk Analysis (IRA) operationalization triad.**

1. Cross-sectoral data sharing and integration
  - Availability, applicability, and use of environmental monitoring data
  - Data sharing and reporting arrangements
  - Cross-sectoral risk mapping
  - Technologies
  - Policies
2. Stakeholder inclusion and holistic engagement
  - Communities
  - Decision-makers
  - Other relevant sectors
3. Resource mobilization and allocation
  - Prioritization
  - Alignment of sectoral priorities
  - Availability of pooled funds

### Essential components of integrated risk analysis

As part of the lessons learned from the JRA OT training and operationalization, study participants expressed the need for a more integrated process for risk assessments of environmentally driven biological threats that would enable cross-sectoral data sharing for risk characterization; better inclusion and engagement of the environment and other relevant sectors (including the Kenya Meteorological Department under the Ministry of Environment, Climate Change and Forestry, and the National Environment Management Agency), decision makers, and community representatives; and proactive resource mobilization and allocation for joint planning and implementation of risk mitigation and communication interventions. We describe these components as the “core IRA operationalization triad” (*Panel 2*).

**Fig 1:**
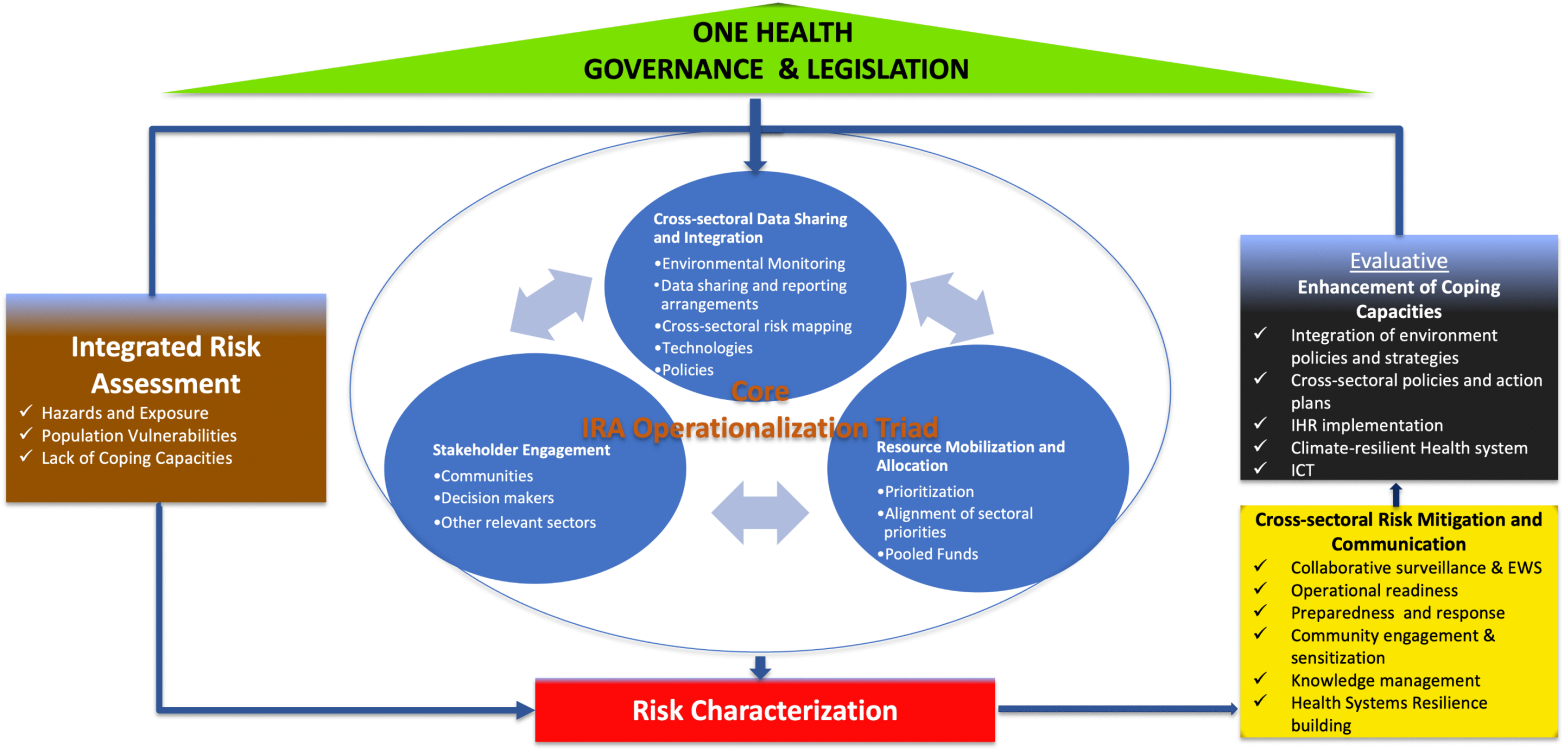
Integrated risk analysis framework.

In addition to findings from the KI interviews, a primary outcome of the stakeholder validation workshop was agreement on the importance of the core IRA operationalization triad as essential components of the newly developed IRA framework (Fig 1). There also was consensus on the need for better OH governance and legislation enactment as an overarching component of the framework, to enable better integration of the environment sector into the implementation of OH activities.

As part of an interactive scenario planning session, workshop participants presented applicable cross-sectoral data for risk analysis of environmentally driven EIDs using the examples of CCHF, MVD, and MERS. A final list of variables by category (epidemiological, environmental, socio-anthropogenic, climatological, and socioeconomic) were collated as an output of the group work discussions on addressing IRA data gaps. These findings are provided in Tables A, B, and C of the attached supporting information (S1 Appendix).

Based on the overall study findings, we present a summary of the key themes that emerged from the study as essentials components of the IRA framework.

**i) Improving cross-sectoral data sharing and integration**: Participants regarded the issues around accessing and sharing cross-sectoral data as a major challenge hindering collaboration of the animal and human health sector with the environment sector. Some specific issues highlighted by KIs include limited knowledge about relevant environmental variables required to improve risk characterization, lack of policies mandating the sharing of data across sectors, lack of data integration due to different reporting platforms, and the need to scale up the operationalization of OH at the county levels to enable cross-sectoral data integration.

*“I remember with El Nino that we had contacted the environment ministry and UNEP. We wanted to have a meeting where they could help us to make our risk map better and to tell us, because of the topography of some of these places, if there are high risks of flooding and all that. We didn’t really get to get that information and, again, it’s because the environment has always been a weak link but things are improving.” –* National A Participant

*“We don’t know where to get this data actually. Not even knowing where to get the data, the environment person is unknown.” –* County E Participant

*“We need a platform, and on that platform, we really need to agree on what we are going to monitor. And what would be critical is to have this monitoring within our national legislation. So, when I’m doing license conditions, when I’m giving standards, I’m using standards that are based on these parameters that need to be monitored.”* – National D Participant

*“There’s a lot of unknown in the environmental sector. And if we knew what kind of data could be pulled and from who, I think we would support–* Research Partner D1

**ii) Early stakeholder inclusion and holistic engagement:** KIs highlighted the need for better inclusion of stakeholders including decision makers, communities, and other relevant sectors as part of IRA processes for buy-in and support. Holistic engagement of stakeholders was also considered crucial for risk-informed joint decision-making and for the implementation of community-level risk mitigation and communication interventions.

*“The decision makers are not aware of this concept, and they actually don’t understand the benefit of having this thing in place.” –* County E Participant

*“..the knowledge at community level is very important. Then you will speak with the community and understand how they understand this disease first* – Research Partner D2

**iii) Proactive cross-sectoral resource mobilization and allocation**: There was agreement about the need to prioritize resources for conducting JRAs and ensuring alignment in sectoral priorities to enable the availability of sustainable pooled funds for implementation of risk mitigation and communication interventions.

*“Resource allocation is one of the main challenges for management of all these issues or problems and diseases. But now with the JRA, to ensure that there is that allocation of resources at the end of it, is to really onboard the decision makers from the start. The process needs to be initiated by them.”* – National A Participant

**iv) Policies and legislation enactment for addressing OH governance challenges:** Participants emphasized the need for the implementation of national and county OH policies as an overarching component of the IRA framework to enable the successful implementation of the cross-cutting components of the core IRA operationalization triad across the entire spectrum of risk analysis. Participants also highlighted the importance of an evaluation component to ensure regular evaluation of the impact of joint risk-based OH activities for the enhancement of long-term coping capacities.

*“The policy has remained with the national, so maybe if there is a policy that is going to bring us together and work together, then also being supportive of those trainings (we need the trainings), we cascade them down to the other levels as a unit. So that the One Heath* *approach is not just seen at the national and even the county level. It is something that has to drill down to the community.”* – County C Participant

The thematic features of the essential components of the IRA framework based on other empirical study data are outlined as a supporting information (S2 Text).

Minor themes emerging from participant responses included the need to account for socio-economic and socio-anthropogenic factors and lack of coping capacities that increase population vulnerabilities to environmentally driven EIDs as part of risk assessments, to better inform risk characterization.

*“But with frequent droughts, with more and more species diminishing because of drought, people are trying to change their main or their preferred species to camel because they are hardy. And you realize that some communities that never used to keep livestock in the past, I mean, camels, in the past, are now embracing camel-rearing. And then the other issue is the cultural aspect, the kind of attachment communities that keep camels have with their camels.”* – County G Participant

Poverty was also considered as a major socioeconomic factor that contribute to population vulnerability to EIDs.

*“There’s also the issue of poverty by the way in terms of communities. And that’s why I think you find them, because of their poverty levels, they will want even to feast on carcasses. You find that maybe there’s dead cattle somewhere. And because these people maybe, in terms of livelihood support they are disadvantaged, they would want to share the meat out or something, and of course, it supports that transfer.”* – County D Participant

There were some highlighted examples of how frequent population movement facilitates disease spread nationally and within and outside the East Africa region.

*“Every five years we experience election cycles, general election or national elections. So, people move from one region to another, depending on their preferred candidates. Some are registered voters in Nairobi, and some are registered voters in Mombasa, so we expect movement. Then there is also the aspect of people looking for employment, better opportunities for studying and work opportunities. Then we also sometimes, every five years, we experience challenges in terms of conflict. So, there is that conflict between human and livestock. There’s a human-animal conflict. Then there is a conflict between, ethnic conflict that causes people to move.”* – County E Participant

*“There is that movement for international trade. An animal could be here today, and the next time, you’ll find it in the Middle East or even in Ethiopia.”* – County G Participant

Highlighted example of lack of coping capacities included limited laboratory diagnostics capacities and low country capacities for risk reduction.

*“There’s issue of the laboratory. So, for example, like in health, at least we need to have some of the labs that we can test.”* – County A Participant

*“Where we are sitting is the largest laboratory, but again we might not be having enough reagents or all the reagents to test what we might have suspected.”* – County D Participant*.*

*“One of the things that I think again that really has to be borne in mind is you can reduce risk, but it has to be in accordance with the capacities of the countries. That means that in some countries you are going to be able to be better at risk reduction than others because the capacity is very low”* – Partner A (Environment)

Some participants also described the need for identification of cross-sectoral risk mitigation and communication interventions that could be jointly implemented across all applicable sectors and with communities.

*“We can also have something like that where we can just think of a particular disease based on our region and the threats we have, we can think of a particular disease then we try to see how we can jointly address an outbreak. It could also give us that sense of activating the One Health so that now we familiarize from the environment what we need to do from the animal health from the human health, what do we need to do in case.”* – County A Participant

*“I’ve seen community reporting being very efficient in early warning systems.* *And also asking farmers or seed groups, for example, asking them. For them reporting, it is a basic thing like abortions in animals. A basic thing that is flooding, yeah, basic things like deaths, when they start seeing livestock deaths. Yeah, that’s community reporting. It should be encouraged for early warning, early warning for zoonotic diseases., To me it’s a good way of tackling this issue of early warning. Involving the community in reporting.”* – County F Participant

## Discussion

Our exploratory study addresses the underexplored topic of climate-induced cascading pandemic risk drivers and the importance of streamlining environment sectoral priorities into the operationalization of the OH approach. A prominent feature of participant responses is the need to improve cross-sectoral engagement of the environment sector with the animal and human health sectors on OH activities in Kenya, in line with calls for a more integrated approach to OH [24]. Our study findings align with recommendations for adopting data-driven risk analysis-based strategies to identify geographic areas where environmentally driven EIDs are likely to emerge and to characterize potential risks of transmission to inform the implementation of risk reductions strategies [17,25].

The risk dimensions adopted under the newly developed IRA framework account for environmental drivers, population vulnerabilities and a systemic lack of coping capacities that increase outbreak exposure risks, in accordance with the INFORM GRI and other related work. [19,26,27]. Our study findings help address gaps in existing assessment methodologies and contribute to knowledge about some of the environmental drivers of EIDs in Kenya. We utilized findings from an integrative review study on improving risk analysis of the environmental drivers of CCHFV, MVD, and MERS in the East Africa region to form the basis of the initial conceptualization of the IRA framework under this study [20].

Important insights emerged from our qualitative case study about the JRA operationalization process in Kenya. A key distinction between the JRA process, a curriculum-based, 10-step process focused on the process of conducting joint risk-based activities across sectors, and the IRA framework described in Fig 1 is in the use of the JRA OT for addressing specific health threats including cross-border outbreaks. In 2022, for example, the JRA OT was operationalized in four Western counties included in the study (Busia, Bungoma, Kakamega, and Vihiga) to jointly assess priority zoonotic diseases and risk of introduction of Ebola Virus Disease (EVD) into Kenya from Uganda following the start of the EVD outbreak in September 2022 [28].

Like the JRA process, the IRA framework model promotes the involvement of different stakeholders across relevant sectors and goes a step further by highlighting the importance of holistically engaging communities as key stakeholders in IRA and JRA processes. However, in contrast to the JRA process, the IRA framework model utilizes a multihazards scenario approach that incorporates environmental parameters into risk models. As a data-driven risk-based framework that enables the use of both quantitative and qualitative data in the risk models, the IRA framework adopts environmental monitoring techniques and systematically accounts for risk-based measures across the entire spectrum of risk analyses processes. These measures include eco-epidemiological, socio-economic and other cascading risk factors that facilitate the spillover, emergence/reemergence, and spread (SES) of EIDs as an essential component of IRA in relation to the need for improving cross-sectoral data sharing and integration. Inclusion of these variable types under the IRA model is critical for informing data-informed risk mitigation and communication interventions, proactive resource mobilization, and implementation of future environmentally driven spillover and outbreak prevention strategies.

Another distinction of the IRA framework from the JRA OT is the inclusion of an evaluation component that describes essential factors to consider in evaluating the level of enhanced coping capacities due to IRA processes. Addition of this component under the IRA framework aligns with policy recommendations for improving OH risk governance and regulations. Overall, the IRA framework model utilizes both a cross-sectoral and multihazards scenario approach that integrates risk modeling data (in addition to surveillance data and qualitative data) and environmental parameters into risk models. Based on the insights that emerged from our study, we consider findings from the JRA exercises to be a complementary source of qualitative data under the IRA framework model.

Findings from this exploratory study complement prior work on identifying the most relevant environmental monitoring, climatological, and socioecological variables that could be used to improve risk characterization and for the implementation of appropriate cross-sectoral risk mitigation and communication interventions [20,29]. Identified risk-based interventions under the IRA framework model are aligned with the WHO Health EDRM Framework. The Health EDRM framework focuses on assessing, mitigating, and communicating risks across different inflection points and along the continuum of disaster prevention and risk management including preparedness, operational readiness, response, and health systems and community resilience building [23].

### Policy implications

The policy implications of our study findings relate to the need for the development and implementation of national and sub-national OH policies in Kenya to improve the operationalization of OH and to enable the establishment of functional COHUs across the country’s 47 counties. Study findings may also contribute to strengthening existing environmental sector policies and legislations to ensure alignment of sectoral priorities and to improve the governance of biological risk reduction and mitigation activities. There is an opportunity to utilize our findings to inform revisions to relevant environmental policies and social and governance standards using a OH approach.

A feasible OH policy option that emerged from our findings is to incorporate the risk analysis of environmental drivers of zoonotic biological threats (in relation to SES) as a mandatory part of environment impact assessments. This approach to IRA may serve as a mechanism for addressing shared commonalities across sectors, in line with similar recommendations from previous assessment studies [30,31]. Adopting a risk-based approach to EID prevention and control, and policymaking and implementation represents the required shift from a reactive outbreak response to a proactive pandemic prevention and health systems resilience building approach.

Overall, our study contributes to informing the current work of the Quadripartite under the Joint Plan of Action (Action track 6) to better integrate the environment into OH [32].

### Recommendations for improving risk analysis processes and ensuring better integration of the environment sector into the One Health Approach

Based on our study findings, we present three key recommendations to enable routine operationalization of IRA processes (including JRAs) as an integral part of the One Health approach in Kenya:

i) Implementation of the cross-cutting components of the core IRA Operationalization Triad (cross-sectoral data sharing and integration, early stakeholder inclusion and holistic engagement, and proactive resource mobilization and allocation) across the entire spectrum of risk analysis.
ii) Inclusion of an overarching component on improving OH governance and legislation enactment to enable risk-based and cross-sectoral data driven decision making for pandemic prevention.
iii) Integration of an evaluative component under the IRA operational framework to track progress with the implementation of cross-sectoral risk mitigation and communication interventions. Inclusion of evaluation metrics under the framework will contribute to the long-term enhancement of institutional and infrastructural coping capacities to prevent and deal with future environmentally driven zoonotic biological threats with epidemic and pandemic potential.

### Limitations

A limitation of the study was that due to limited budget, only half of the study participants were invited to the stakeholder validation workshop to validate the study findings. However, the team balanced out the list of workshop invitees across sectors, areas of expertise and institutional levels to ensure proper representation of study participants. With permission, the primary investigator also followed up with some KIs who were unable to attend the workshop to clarify/request additional information and documents related to the study findings. It is worth noting that the limited number of nominated female participants who met the study selection criteria shed light on a broader issue regarding the need for a balanced gender representation in the operationalization of the OH approach to better address gender-related vulnerabilities to infectious diseases. However, the study team acknowledges that the observations around the gender imbalance is beyond the scope of this study and could help inform future efforts.

Although our findings are largely unique to the Kenyan context, we also obtained useful insights on experiences of some participants who had been engaged in OH implementation and/or the JRA OT operationalization process in other countries. Consequently, study findings on the essential components of IRA processes likely are transferable to other settings that may face similar challenges with cross-sectoral implementation of risk-based OH activities.

## Conclusion

Increasing frequency of EWEs and heightened risks of environmentally driven EID outbreaks of zoonotic origins in Kenya call for routine operationalization of cross-sectoral OH activities such as JRAs using a more integrated approach to risk analysis. As part of EID risk assessments, there is a need to better account for, and integrate knowledge about the environmental drivers of pathogen emergence and/or reemergence that mediate pathogen/disease SES. Risk dimensions should also consider population vulnerabilities and lack of coping capacities that further facilitate disease transmission across the environment-animal-human exposure interface.

In our study, we learned from the experiences of KIs on the JRA OT operationalization in Kenya and solicited KI perspectives on essential components of an IRA operational framework for addressing environmentally driven EIDs. Our study findings highlight the need for improving cross sectoral data sharing and integration, early stakeholder inclusion and holistic engagement, proactive cross-sectoral resource mobilization and allocation, and enactment of OH policies and legislations as critical components of the IRA framework. These findings have been used to finalize the development of the framework for improving risk analysis processes and to ensure better integration of the environment sector into the implementation of OH activities for pandemic prevention.

## Supporting information

S1 Text Semi-structured key informant interview guide. (DOCX)

S2 Text Thematic features of the essential components of IRA. (DOCX)

S1 Table Thematic framework analysis template. (DOCX)

S1 Appendix Stakeholder validation workshop findings. (DOCX)
