# Supplementary material for: Improving risk analysis of environmentally driven zoonotic biological threats as a primary pandemic prevention approach: A case study of the Tripartite Joint Risk Assessment Operational Tool operationalization in Kenya
Source: PLOS Glob Public Health. 2026 Jul 1;6(7):e0006560. doi: 10.1371/journal.pgph.0006560 (PMC13322548; doi:10.1371/journal.pgph.0006560)
Supplement: S1 Text — (DOCX) [file pgph.0006560.s001.docx]

**S1 Text. Semi-structured Key Informant Interview Guide**

*(Version 1)*

Improving Risk Analysis of Environmentally Driven Zoonotic Biological Threats as a Primary Pandemic Prevention Approach: A Case Study of the Tripartite Joint Risk Assessment Operational Tool (JRA OT) Operationalization in Kenya

This semi-structured interview guide has been developed to learn from the experiences of key informants with the JRA OT operationalization process and to solicit feedback on how to improve integrated risk analyses of environmentally driven emerging and/or reemerging zoonoses at the environment-animal-human exposure interface, to inform cross-sectoral decision making for pandemic prevention. The included questions are intended to serve as a framework to guide the discussions and includes the use of prompts and probes to obtain additional information from participants for the purpose of addressing the study aim. In some instances, these questions may be asked in a different sequence than the sequence outlined in the interview guide thus allowing for the interviewer to diverge slightly from the guide. It is also expected that other relevant questions will be asked of study participants based on the initial responses provided to some of the identified questions below.

**Greetings**

Thank you for taking out time to participate in this interview session. I would like to start by reiterating that everything shared with me during this session is confidential and no identifying information (such as your name or job title) will be linked or used in any report associated with this study. I will be recording the interview session solely for the purpose of staying engaged in our conversations and following your responses closely over the course of the interview session to limit the distractions from extensive notetaking. Afterwards, the study team will transcribe the interviews for use in our data analysis, which would entail identifying themes and main ideas that emerge from the interview data. This information will be used to help address our study aim and objectives of learning from your experiences with the JRA OT institutionalization in Kenya and obtaining your professional expertise to finalize the development of an integrated risk analysis framework.

Before we begin the interview, I would also like to provide you with ample opportunity to ask any questions or concerns about the interview and the overall study aim. Do you have any questions?

**Introduction**

1. Can you tell me about your background including your current job and the role you serve?
   1. How long have you worked in this role?
   2. How does your current job relate to the expertise you bring to the Tripartite JRA OT operationalization process?
2. Can you briefly walk me through the type of training you received on the use of the JRA OT for characterizing the risk of biological threats at the national and/or sub-national level?
   1. If the training met your expectation, can you tell me more about some ways the training met your expectations?
   2. If the training did not meet your expectation, can you tell me more about this?
   3. Based on your experiences with past trainings, in what ways do you think you would most benefit from future related cross-sectoral trainings?

**Joint Risk Assessment Process**

1. What has been your experience working across different stakeholder groups on the risk assessments of zoonotic biological threats using the JRA OT?
   1. Which sectors were represented in the risk assessment of specific biological threats you were involved in?
   2. How was consensus reached across different stakeholder groups to characterize the level of risk?
   3. How was the level of uncertainty considered for the risk characterization of a given outbreak under assessment?
2. What has been your experience carrying out the risk pathway analysis for each identified zoonotic biological threats?
   1. In what ways do you think this process could be improved?
3. What other experiences have you had with conducting cross-sectoral/joint risk assessments using other types of risk assessment processes/tools?
   1. Can you tell me more about this?

**Understanding the Environment-infectious disease interface**

Research shows that since the turn of the century, there has been a rise in the frequency and severity of emerging and reemerging zoonotic infectious disease outbreaks due to environmental changes.

1. In your opinion, what do you consider to be key environmental drivers of the emergence/reemergence of zoonotic biological threats?
   1. You mentioned x environmental drivers. Which of these identified environmental drivers do you consider to be specific to the Kenyan country context?
   2. What would you consider to be environmental factors that drive the spillover, emergence, and spread of CCHFV, MARV, and MERS-CoV?
   3. What communities/population groups in Kenya are most at-risk of outbreaks of EIDs such as CCHF, MVD, and MERS?
   4. In general, what are some population vulnerabilities that increase the risk of EID emergence/reemergence?
   5. What are some factors that contribute to a systemic lack of coping capacities for addressing spillover, emergence, and spread of EIDs in Kenya?
2. How can environmental monitoring data be utilized for integrated risk analysis processes?
   1. What is your experience with utilizing environmental data as part of the joint risk analysis processes?
   2. What type of technologies are needed to improve the use of environmental monitoring data as part of integrated risk analysis processes?
   3. What policies are in place to enable the use of environmental data as part of EID risk analysis processes?
   4. Can you tell me more about the risk mapping process for EIDs in Kenya such as CCHF, MVD, and MERS?
   5. What are some challenges that need to be addressed to enable the successful integration of environmental data with animal and human health epidemiologic data?
   6. Can you tell me more about other cross-sectoral data that you consider relevant for assessing the risk of environmentally driven zoonotic EIDs to inform the development and implementation of risk management and risk communication options?
3. In your opinion, what are essential risk components that must be accounted for to ensure an early and effective response to environmentally driven EIDs at the source?
4. How are resources mobilized and allocated to ensure an early and effective response to a biological threat following the JRA process?
   1. In your opinion, what actions are required to ensure a systematic allocation of resources for the implementation of cross-sectoral risk management and risk communication interventions following an EID risk characterization?

**Improving Community Engagement to ensure an early and effective response to EIDs**

1. How do you think we can better engage communities in the early warning response to the emergence/reemergence of zoonotic EIDs of epidemic and pandemic potential?
   1. What has been your experience with the involvement of community representatives on the JRA OT operationalization process?
   2. In what ways do you consider community engagement processes to be different in accounting for environmental factors associated with zoonotic biological threats?

**Ensuring better integration of the Environment sector in the One Health Approach**

1. What ways do you think the environment sectors can be better engaged with the human and animal health, and other relevant sectors in the early warning and response to EIDs?
   1. What strategies do you believe are effective for better integrating environment sectoral strategies into the One Health approach to reduce the risk of zoonotic EIDs of epidemic and pandemic potential?

**Decision-making to enhance Pandemic Prevention, Preparedness and Response**

1. How are decisions taken to ensure appropriate representation of relevant stakeholder groups in the risk management and risk communication strategies recommended for implementation following the JRA process?
   1. How was consensus reached across different stakeholder groups in identifying appropriated risk management and risk communication recommendations following the risk characterization process?
   2. In what ways do you think collaborative decision-making process can be improved with the environmental sector?
   3. What policies have been developed and implemented in support of identified risk management and risk communication recommendations following the JRA process?
   4. What effect has the JRA Operationalization process had on cross-sectoral coordination, collaboration and communication for decision making?
   5. What do you consider to be critical actions that must be taken by decision makers to mitigate the threats posed by EIDs and to reduce population vulnerabilities to future threats?
2. How do you think that the JRA OT risk pathway analysis and risk characterization process has impacted the level of preparedness of Kenya to better address environmentally driven zoonotic biological threats?
   1. In what ways do you think risk analysis processes can be improved to better account for environmental drivers of zoonotic EIDs and other biological threats?
   2. Can you tell me more about some early warning mechanisms adopted in response to EID threats in Kenya?

**Policy Making and Implementation**

1. What are some best practices adopted for operationalizing the JRA OT for the assessment of zoonotic biological threats in Kenya at the national and/or sub-national level?
   1. What factors were considered in the institutionalization of the JRA for the assessment of zoonotic biological threats?
2. Can you walk me through some of the policies that have been implemented as a direct (or indirect) consequence of the JRA OT Operationalization in Kenya (at national and sub-national level?
   1. What are your opinions on the effectiveness of any of these policies?
   2. What are your opinions on the gaps in these policies, if any?
3. In your opinion, what type of additional policies are required to address environmentally driven EIDs like CCHF, MVD, and MERS?
   1. In your opinion, what is required to improve operational readiness to ensure an effective cross-sectoral rapid response to outbreaks of EIDs?
4. What type of environmental policies are required to mitigate impacts of environmental changes like climate and land use changes on increasing risks of zoonotic disease emergence/reemergence in the region?

**Closing Questions/Interview wrap-up**

1. Before we end the interview, is there any additional information you would like to add about your experience coordinating and working across different sectors on the JRA OT operationalization at the national and/or sub-national level?
   1. Is there anything you might do differently in the future while conducting the risk pathway analysis under the JRA OT in assessing the risk of a given biological threat? If yes, what would you do differently?
   2. What is a final message you have about what is required to improve cross-sectoral decision making for pandemic prevention and to prevent future spillover of environmentally driven biological threats?

Thank you for your time and participation in this interview. Please feel free to reach out to me at any time if you would like to discuss further. If this is okay with you, we might also contact you later to clarify some information as part of our validation process. Do let me know if you will be available to be contacted again for clarification purposes, if needed.

*(Version 2 for interviews with participants from the Quadripartite Alliance)*

This semi-structured interview guide has been developed to learn from the experiences of key informants with the JRA OT operationalization process and to solicit feedback on how to improve integrated risk analyses of environmentally driven emerging and/or reemerging infectious diseases (EIDs) of zoonotic origins at the environment-animal-human exposure interface, to inform cross-sectoral decision making for pandemic prevention. The outlined questions below are intended to serve as a framework to guide the discussions and includes the use of prompts and probes to obtain additional information from participants for the purpose of addressing the study aim. In some instances, these questions may be asked in a different sequence than the sequence outlined in the interview guide thus allowing for the interviewer to diverge slightly from the guide. It is also expected that other relevant questions will be asked of study participants based on the initial responses provided to some of the identified questions.

**Greetings**

Thank you for taking out time to participate in this interview session. I would like to start by reiterating that everything shared with me during this session is confidential and no identifying information (such as your name or job title) will be linked or used in any published report associated with this study. I will be recording the interview session solely for the purpose of staying engaged in our conversations and following your responses closely over the course of the interview session, to limit the distractions from extensive notetaking. Afterwards, the study team will transcribe the interviews for use in our data analysis, which would entail identifying themes and main ideas that emerge from the interview data. This information will be used to help address our study aim and objectives of learning from your experiences with the JRA OT institutionalization in Kenya and obtaining your professional expertise to finalize the development of an integrated risk analysis framework for addressing the spillover, emergence, and spread of environmentally driven EIDs.

Before we begin the interview, I would also like to provide you with ample opportunity to ask any questions about the interview and the overall study aim. Do you have any questions?

**Introduction**

1. Can you tell me about your background including your current job and the role you serve?
   1. How long have you worked in this role?
   2. How does your current job relate to the expertise you bring to the Tripartite JRA OT operationalization process?
2. Can you briefly walk me through the type of training provided on the use of the JRA OT for characterizing the risk of biological threats at the national and/or sub-national level?
   1. If the training met your expectation, can you tell me more about some ways the training met your expectations?
   2. If the training did not meet your expectation, can you tell me more about this?
   3. Based on your experiences with past trainings, in what ways do you think stakeholder would most benefit from future related cross-sectoral trainings?

**Joint Risk Assessment Process**

1. What has been your experience working across different stakeholder groups on the risk assessments of zoonotic biological threats using the JRA OT?
   1. Which sectors were represented in the risk assessment of specific biological threats you were involved in?
   2. How was consensus reached across different stakeholder groups to characterize the level of risk?
   3. How was the level of uncertainty considered for the risk characterization of a given outbreak under assessment?
   4. What are some key lessons that have emerged from the JRA process in Kenya?
2. What has been your experience carrying out the risk pathway analysis for each identified zoonotic biological threats?
   1. In what ways do you think this process could be improved?
3. What other experiences have you had with conducting cross-sectoral/joint risk assessments using other types of risk assessment processes/tools?
   1. Can you tell me more about this?

**Understanding the Environment-infectious disease interface**

Research shows that since the turn of the century, there has been a rise in the frequency and severity of emerging and reemerging zoonotic infectious disease outbreaks including outbreaks due to environmental changes.

1. In your opinion, what do you consider to be key environmental drivers of the emergence/reemergence of zoonotic biological threats (using specific examples)?
   1. You mentioned x environmental drivers. Which of these identified environmental drivers do you consider to be specific to the Kenyan country context?
   2. What would you consider to be environmental factors that drive the spillover, emergence, and spread of CCHF, MVD, and MERS-CoV-2?
   3. What communities/population groups in Kenya are most at-risk of outbreaks of EIDs such as CCHF, MVD, and MERS-CoV-2?
   4. In general, what are some population vulnerabilities that increase the risk of EID emergence/reemergence and spread?
   5. What are some factors that contribute to a systemic lack of coping capacities for addressing spillover, emergence, and spread of EIDs in Kenya?
2. How can environmental monitoring data be utilized for integrated risk analysis processes?
   1. What is your experience with utilizing environmental data as part of the joint risk analysis processes?
   2. What type of technologies are needed to improve the use of environmental monitoring data as part of integrated risk analysis processes?
   3. What policies are in place to enable the use of environmental data as part of EID risk analysis processes?
   4. Can you tell me more about the risk mapping process for EIDs such as CCHF, MVD, and MERS (or other examples) in Kenya?
   5. What are some challenges that need to be addressed to enable the successful integration of environmental data with animal and human health epidemiologic data?
   6. Can you tell me more about other cross-sectoral data that you consider relevant for assessing the risk of environmentally driven zoonotic EIDs to inform the development and implementation of risk management and risk communication options?
3. In your opinion, what are essential risk components that must be accounted for to ensure an early and effective response to environmentally driven EIDs at the source?
4. How are resources mobilized and allocated to ensure an early and effective response to a biological threat following the JRA process?
   1. How do partner organizations work with the government on resource allocation and mobilization required to implement recommended interventions identified following the JRA process?
   2. In your opinion, what actions are required to ensure a systematic allocation of resources for the implementation of cross-sectoral risk management and risk communication interventions following an EID risk characterization?

**Improving Community Engagement to ensure an early and effective response to EIDs**

1. How do you think we can better engage communities in the early warning response to the emergence/reemergence of zoonotic EIDs of epidemic and pandemic potential?
   1. What has been your experience with the involvement of community representatives on the JRA OT operationalization process?
   2. In what ways do you consider community engagement processes to be different in accounting for environmental factors associated with zoonotic biological threats?

**Ensuring better integration of the Environment sector in the One Health Approach**

1. What ways do you think the environment sectors can be better engaged with the human and animal health, and other relevant sectors in the early warning and response to EIDs?
   1. What strategies do you believe are effective for better integrating environment sectoral strategies into the One Health approach to reduce the risk of zoonotic EIDs of epidemic and pandemic potential?

**Decision-making to enhance Pandemic Prevention, Preparedness and Response**

1. How are decisions taken to ensure appropriate representation of relevant stakeholder groups in the risk management and risk communication strategies recommended for implementation following the JRA process?
   1. How was consensus reached across different stakeholder groups in identifying appropriated risk management and risk communication recommendations following the risk characterization process?
   2. In what ways (if any) are decision makers involved in the JRA operationalization process?
   3. In what ways do you think collaborative decision-making process can be improved with the environmental sector?
   4. What policies have been developed and implemented in support of identified risk management and risk communication recommendations following the JRA process?
   5. What effect has the JRA Operationalization process had on cross-sectoral coordination, collaboration and communication for decision making?
   6. What do you consider to be critical actions that must be taken by decision makers to mitigate the threats posed by EIDs and to reduce population vulnerabilities to future threats?
2. How do you think that the JRA OT risk pathway analysis and risk characterization process has impacted the level of preparedness of Kenya to better address environmentally driven zoonotic biological threats?
   1. In what ways do you think risk analysis processes can be improved to better account for environmental drivers of zoonotic EIDs and other biological threats?
   2. In what ways do you think data gaps can be addressed to improve the effectiveness and level of accuracy of the risk pathway analysis and risk characterization process?
   3. Can you tell me more about some early warning mechanisms adopted in response to EID threats in Kenya?

**Policy Making and Implementation**

1. What are some best practices adopted for operationalizing the JRA OT for the assessment of zoonotic biological threats in Kenya at the national and/or sub-national level?
   1. What factors were considered in the institutionalization of the JRA for the assessment of zoonotic biological threats?
   2. What has been the level of involvement of different counties in the JRA operationalization?
2. Can you walk me through some of the policies that have been implemented as a direct (or indirect) consequence of the JRA OT Operationalization in Kenya (at national and sub-national level?
   1. What are your opinions on the effectiveness of any of these policies?
   2. What are your opinions on the gaps in these policies, if any?
3. In your opinion, what type of additional policies are required to address environmentally driven EIDs like CCHF, MVD, and MERS?
   1. In your opinion, what is required to improve operational readiness to ensure an effective cross-sectoral rapid response to outbreaks of EIDs?
4. What type of environmental policies are required to mitigate impacts of environmental changes like climate change and land use changes on increasing risks of zoonotic disease emergence/reemergence in the region?

**Closing Questions/Interview wrap-up**

1. Before we end the interview, is there any additional information you would like to add about your experience working with the government across different sectors in coordinating the JRA OT operationalization at the national and/or sub-national level?
   1. Is there anything you might do differently in the future while conducting the risk pathway analysis under the JRA OT in assessing the risk of a given biological threat? If yes, what would you do differently?
   2. What are your closing remarks on how to prevent future spillover, emergence and spread of environmentally driven biological threats?
   3. What is a final message you have about what is required to improve cross-sectoral decision making for pandemic prevention and

Thank you for your time and participation in this interview. Please feel free to reach out to me at any time if you would like to discuss further. If this is okay with you, we might also contact you later to clarify some information as part of our validation process. Do let me know if you will be available to be contacted again for clarification purposes, if needed.
