# Supplementary material for: Improving risk analysis of environmentally driven zoonotic biological threats as a primary pandemic prevention approach: A case study of the Tripartite Joint Risk Assessment Operational Tool operationalization in Kenya
Source: PLOS Glob Public Health. 2026 Jul 1;6(7):e0006560. doi: 10.1371/journal.pgph.0006560 (PMC13322548; doi:10.1371/journal.pgph.0006560)
Supplement: S2 Text — (DOCX) [file pgph.0006560.s002.docx]

**S2 Text. Thematic Features of the Essential Components of an Integrated Risk Analysis Framework Based on Study findings**

| **Theme** | **Description** | **Cases (# of Participants)** | **Counts (# of Empirical Data/Quotes)** | **Example of Empirical Data Evidence across different stakeholder groups** |
| --- | --- | --- | --- | --- |
| 1. **Improving Cross-sectoral Data Sharing and Integration** | This theme addresses KI views on the need to have in place data sharing arrangements and platforms for reporting and sharing relevant environmental monitoring and risk mapping data with the animal, wildlife, and human health sectors. It also covers the policies and technologies that are required to enable cross-sectoral data sharing and integration to inform risk characterization and data-driven decision making for pandemic prevention | 28 | 354 | *“There was some issue especially the issue of the platform system for the reporting. They could not access our report. Likewise, we could not access their report, that's very key now because without the reports decision making becomes a problem.”*- County A Participant  *One of the challenges was having data that would inform why we think a certain pathway is the highest risk and also a lot of the assumptions was that the data is the right one or we had the right data. We had to do a lot of literature review at that point and when we do not have the expert within the team, who would have this information, it becomes even more difficult.”* - National A (One Health)  *“I mean, almost everybody you'll probably speak to, they would agree that we need better data sharing between the human health world, the veterinary world, and the environmental world. But the reality is, these are complete silos. When I work on biodiversity, I either get my permit from the Ministry of Environment. Or if it's the same ministry or a different one, from the Park Management Authority. That's pretty clear, but I have no permission to sample human health.I mean, it's the same as trying to look at climate change, and climate change is cross-sectoral and it has health implications, but also, economy should play in, environment, agriculture. I think, with health, it should be the same.”*- Partner A (Environment)  *“Environment also have data, rarely do we see that data. That data has never been brought together, so we are struggling a lot. Maybe the data that will have looked at the environment will have informed us on what needs to be done early to avoid some of the diseases. The data that would have been in veterinary would also have informed us when are we supposed to act or take action."* - County C Participant  *"Everyone is going electronic. People are trying to make sure that data goes electronic and I think we need to try invest within that space. For example, our government, sometimes I feel that they do not optimize the usage of open source freely available data sets, for example, set data. We've not certainly utilizing those, and therefore I feel, one, we need to have electronic systems, and two, we need to start interrogating some data sets that we've traditionally not made full use of, for example, satellite imagery."* - Partner D (Infectious Disease Research)  *“If you just say, “Oh, it's cross-sectoral, you have to work together,” it will never work. But give them a chance to develop their model, and at the end, bring it together and say, “Oh, here is an overlap but you have a problem. There are trained system modelers. And I think data is one aspect we can actually focus on, as part of it, because all sectors do something with their data. And so, it's really around using the models, understanding the models from the various sectors and using the models together.” –* Partner A (Environment)  *“We should be able to address the issue of data sharing because when you come up with one health policy for the country, what does it say about, or what we did say about data -sharing and what data do they want to share. So that should be handled, and I think it's one of the things that we are thinking perhaps we'll be able help us cure some of challenge.”* - National A (One Health)  *“I think one of the challenges is, one, the access and availability of environmental data. Most of the time, the custodian of the environmental data, the Kenyan Meteorological Department, when we talk of, maybe, weather data, the climatic data in terms of temperature as moisture, precipitation, and all that. And in most cases, when we think of the representation of the environment in joint risk assessment, we talk of public health environmentalists. We rarely think of the Kenyan Meteorological Department, and they are the custodians of this kind of data. So, the access and utilization sometimes becomes a challenge.”* – County G Participant  *“What you are asking goes beyond the JRA. The JRA might help to pop up where there are gaps in terms of data. And remember that why I said for a decision maker, he needs to connect with a big picture. The big picture here is maybe you have an anthrax preparedness and response plan. So, you will say, boss, the risk is high, but the uncertainty is also high. Why? Because the data we don't have, we have very little in terms of environmental data. We made some assumptions. So, we need to conduct research, or we need first to maybe look at if maybe those who came didn't bring those data. But let's talk, we know, let's if in the case they are not available, we need to the country need to conduct some study.”* – Partner B (One Health)  *“There's a lot of unknown on the environmental sector. And if we knew what kind of data could be pulled and from who, I think we would support. But for us, we would probably be interested in things like climate change, data, things like vegetation cover data. Over time, things like now the changing land use, for example, 10 years ago in this area, there were no this and that. Now we have 50 percent occupied by settlements. And now we have quarries everywhere. So, for us, that is data that is relevant for our EID kind of research. But we don't know who the custodian is. We don't know how we would get it if we wanted it, because we don't know the person who collects, if at all it's collected. So that would be the kind of information that we are interested in.”* - Partner D (Infectious Diseases Research)  *“In terms of sharing, again, the biggest challenge has been the issue of we collect so much data. Which data do you want to see? So, if there's a platform that enables us to see relevant data from the vet, the vet can see relevant data from the human health. And we can also see relevant data from the environment side, not necessarily from the point of collection. But maybe even if I wanted to say, because the DVS does surveillance on avian flu, so if there's a report of increased flu-like symptoms, for example, in Nakuru County, I want to be able to go into some database of sorts and compare to the vet side and see, are there any signals that are coming from the Nakuru side? I'm not interested in all his data.*” – Partner D (Infectious Diseases Research)  Quotes on Environmental Drivers  *“Let's say we have floods, meteorologists have advised us on them, that we shall be experiencing flood or heavy rainfall ahead of us. Now, with joint risk assessment, we shall be able to put in place mitigation measures with regards to flood.”* - County F Participant  *“There was one season that elephants moved all the way from Tsavo to Tana River, and they actually caused a lot of havoc. So, if there's that possibility of migration patterns, also that risk of transmission of pathogens*.” – County E Participant  *“We would need things like meteorological data. I think that would be useful. We would also need things about landscape changes, if there are any plants to say have people, maybe a certain part of the forest is being changed to agricultural use. So such things would be useful.”* – National C (Wildlife)  *“Ofcourse, one would be rainfall data. Rainfall, in the sense that we should understand the amounts of rainfall across the county, issues to do with even temperature variation, that we are talking about, even humidity, because you see, I know the disease vectors thrive at maybe certain temperature. Like you see the COVID, it has been associated with the cold, and then there are those that would want a bit higher temperature, so something like that. Then there is also the extent of degradation.” –* County D Participant  *“The best example for environmental data monitoring and how it would help in risk analysis, the best example to use would be the RVF and of course there is the meteorological data. And then there is the data on looking at areas that are flooding or that have stagnant water, issues of vegetation and all that, making a conducive environment for the vectors. So that would be one of the examples of how such data would be useful. I remember with El nino, that we had contacted the environment ministry and UNEP. We wanted to have a meeting where they could help us to make our risk map better and to tell us because maybe of the topography of some of these places. There are high risks of flooding and all that, although we didn't really get to get that information and again it's because of the environment has already been a weak link but things are improving.”* - National A (One Health)  *“I think it would be useful to have data on the distribution, on abundance of species, right? It's not going to happen, okay? So, because it costs an incredible amount of money to do that and there is no real technological solution.”* – Partner A (Environment)  *“We have not worked with people from the environment, and it's really hard to even know, first of all, what kind of data do they collect. I know they collect weather data, but weather data is like publicly available. If I wanted to pull from the meteorological department, I would go in there and pull. But I guess we would want more than that, because something about the environment is that the change doesn't happen overnight. So, it means it has to be kind of long-term monitoring over time about the environment, about things like vegetation cover, about things like, I guess, soil content, in addition to now the climate change.”* – Partner D (Infectious Diseases Research) |
| 1. **Early Stakeholder Inclusion and Holistic Engagement** | This theme captures KIs responses on the importance of involving key stakeholder like decision makers, community members and other relevant sectors throughout the entire risk analysis process to ensure proper buy-in and support | 28 | 297 | *“There's been a challenge of now trying to cascade the JRA to the county level. The challenge is trying to disseminate this information in terms of education and awareness, capacity building of other county employees and other decision makers. You see, at the level of a director, myself and the two others, we need to cascade it and also escalate it at the level of the county executive. So, my experience is absorption or adoption of this concept of JRA is the problem”. -* County D Participant  *“What I think is that we require adequate information of people from different cadres. We just require adequate information. They are not aware. Some of them are not aware. In fact, the people who lead this country may not be aware. It may be a departmental thing or for us three departments and the rest will refuse. So, we require to have forums where we bring attention of all these other people on board so that they learn and from there they can decide. Otherwise, you will technically stay with your knowledge and probably go with it.” -* County A Participant  *“That's where the community, the knowledge at community level is very important. Then you will speak with the community and understand how they understand this disease first.* *So, when you come to assess the uncertainty associated with the probability or the impact, that's where it becomes very important to know a handy community.” -* Partner E (Human Health)  *“I remember there was an interview I also did with Red Cross, and they were asking about the challenges we are facing in the whole of JRA space. And I was telling them that one of the challenges is on resource allocation. The decision makers are not aware of this concept, and they actually don't understand the benefit of having this thing in place. So that is my experience.”*  *"The risk uncertainty, when we lack some of this expertise, we cannot say certainly that probably when we are carrying out assessment, we have high level of certainty when we’re doing this risk assessment. We still lack some of this vital information from this sector, or we need regular information from this sector, to be able now to give a comprehensive risk assessment report. Because both of these services were devolved and counties don't see the need to hire people in these areas who can be able to support some of these activities. So, when you're going out, these routine activities are a bit tricky in the sense that now since we lack expertise, then every sector is working in silos until there is a health emergency, that's when we come together and be able now to carry a joint operation, but missing some of this expertise like environment, sometimes lab. Then it becomes a challenge. Even the certainty levels of a particular risk." -* County E Participant |
| 1. **Proactive Cross-sectoral Resource Mobilization and Allocation** | This theme describes participant responses about the need for cross-sectoral prioritization of resources, alignment in sectoral priorities, and having pooled funding mechanisms in place for operationalization of IRA processes | 27 | 151 | *“We require some resources that can enable us to move even faster. As of today. we have not adequately had some meaningful work. You can say yeah we have the capacity yes but then how do you move without being funded. So, we couldn't do much, other than sensitization in meetings.” -* County B Participant  *“So, you also have come up with a report and share with our bosses. So, such a report also could follow that [JRA] format but not exactly but at least there was that element. though still it did not bear fruits because at that time when we were dealing, there were no resources out of the county. So it was a bit difficult to implement what we had put as a measure.”* - County D Participant  *“Ofcourse resource allocation is one of the main challenges for management of all these issues or problems and diseases. But now with the JRA I think ... To ensure that there is that allocation of resources at the end of it, is to really on -board the decision makers from the start, but ideally from the tool, the process needs to be initiated by them.” -* National A (One Health)  *“I think it, first of all, has to come with policy making process. If we get involved or if we come up with a policy where environmental aspect is incorporated, everything will go smoothly actually, because we cannot work without policy in place. It's a guiding document that should there be anything, we can go back to the policy, we can always refer to the policy in place. And also, for when we are sourcing funds or mobilizing resources, still, there has to be a policy in place that guides the process. So, environment aspect needs to be incorporated in policy making process.” -* County H Participant |
| 1. **Policies and Legislations for addressing OH Governance Challenges** | This theme describes participant views on the policies required to enable the implementation of OH activities at national and county levels and better integration of the environment sector into the OH approach, and to evaluate the impact of risk-based OH activities | 26 | 244 | *“Yes, so I feel, as counties, we need a policy that will anchor JRA. If we have a policy, then it will sort of become compulsory for us to comply and for departments to follow suit.” –* National A (One Health)  *“For example, if there’s mining, the environment sector is involved because they provide licensing, they provide oversight to check whether there’s degradation. So they know when there’s mining much earlier on before the human health people know. There’s a metric of reporting and they could invite the human health people and say, there’s increased mining activity here and we suppose that people are now venturing into the environment in areas where they’ve never gone before, so you guys need to come in and try this. I think that’s one of the areas where we could have an interface.”*- Partner D (Infectious Disease Research)  *“If we can have NEMA, that is National Environment Management Authority, as one of the stakeholders. Because they are the ones actually, so that they can amend on their policies with regards to environmental impact assessment or environmental audits, or environmental social impact assessment. So, we can have NEMA coming in place and see together where they can amend on their legislation policies and ask to do it, environmental impact assessment. If we can have one of the stakeholders, we can all be able to discuss and ensure that it's incorporated.”-* County H Participant  *“The JRA process usually comes out with some risk recommendations that this is what needs to be done, so this is the risk level, this is the hazard that you anticipate, and this is the likelihood. Once we come up with those, then we really need to -- we usually work on a risk communication, so now communicating that to the various stakeholders; the community, the government practitioners, the research community and so on. I think just the straightforward thing that people need to do to ensure that those recommendations are implemented and we probably have a way to track them. Currently, because the government doesn't have a very good policy around JRA, it's not easy to monitor those recommendations. We write those recommendations, we provide that communication to the various stakeholders, but it's really not a formal communication. So people do not really have a way to track whether anybody follows up to implement those recommendations."*- Partner D (Infectious Diseases Research)  *“People alone, even us like we did a policy mapping at some point to look at One Health. So, there are a number of policies in the human health and animal health space which speak to One Health. But I think we did not see any in the environment.But there was one in the wildlife policy.”* National A (One Health)  *“I think it, first of all, has to come with policy making process. If we get involved or if we come up with a policy where environmental aspect is incorporated, everything will go smoothly actually, because we cannot work without policy in place. It's a guiding document that should there be anything, we can go back to the policy, we can always refer to the policy in place. And also, for when we are sourcing funds or mobilizing resources, still, there has to be a policy in place that guides the process. So, environment aspect needs to be incorporated in policy making process”* - County H Participant |
