# Supplementary material for: Improving risk analysis of environmentally driven zoonotic biological threats as a primary pandemic prevention approach: A case study of the Tripartite Joint Risk Assessment Operational Tool operationalization in Kenya
Source: PLOS Glob Public Health. 2026 Jul 1;6(7):e0006560. doi: 10.1371/journal.pgph.0006560 (PMC13322548; doi:10.1371/journal.pgph.0006560)
Supplement: S1 Table — (DOCX) [file pgph.0006560.s003.docx]

**S1 Table. Thematic Framework Analysis Template**

| Overarching Category | Main code | Sub-codes |
| --- | --- | --- |
| 1. Integrated Risk Assessment | 1.1 JRA OT Experience | 1.1.1 Perception on training received |
|  |  | 1.1.2 Risk Pathway analysis |
|  |  | 1.1.3 Cross-sectoral consensus building |
|  |  | 1.1.4 Best practices |
|  |  | 1.1.5 JRA Impact |
|  | 1.2 Hazards and Exposures | 1.2.1 Emergence/Reemergence of Zoonotic threat |
|  |  | 1.2.2 Environmental drivers |
|  |  | 1.2.3 Climate-related events |
|  | 1.3 Population Vulnerabilities | 1.3.1 Socio-economic |
|  |  | 1.3.2 Movement Patterns |
|  |  | 1.3.3 Vulnerable Groups |
|  |  | 1.3.4 Behavioral |
|  | 1.4 Lack of coping capacities | 1.4.1 Points of entry |
|  |  | 1.4.2 Governance |
|  |  | 1.4.3 Communication |
|  |  | 1.4.4 Health System |
| 1. Zoonotic threat Risk Characterization | 2.1 Risk of Escalation of biological event |  |
|  | 2.2 Risk of Spillover/Emergence/Spread |  |
|  | 2.3 Likelihood of future environmental event |  |
|  | 2.4 Potential magnitude of impact | 2.4.1 Socioeconomic |
|  |  | 2.4.2 Environmental |
|  |  | 2.4.3 Health system |
|  | 2.5 Uncertainties | 2.5.1 Access to information |
|  |  | 2.5.2 Data Quality |
| 1. Integrated Risk Analysis Data | 3.1 Availability of Environmental Monitoring Data | 3.1.1 Utilization of Environmental Monitoring Data |
|  |  | 3.1.2 Applicability of Environmental Monitoring Data |
|  | 3.2 Data Integration | 3.2.1 Sectoral representation |
|  |  | 3.2.2 Data Integration Platform |
|  | 3.3 Data sharing and Reporting arrangements | 3.3.1 Informal arrangements |
|  |  | 3.3.2 Formal arrangements |
|  |  | 3.3.3 Data Governance |
|  | 3.4 Risk Mapping data | 3.4.1 Utilization for decision-making |
|  | 3.5 Technologies | 3.5.1 Types of technology |
|  |  | 3.5.2 Capacities for utilization |
|  | 3.6 Availability of Policies | 3.6.1 Implementation of Policies |
|  |  | 3.6.2 Enforcement of Policies |
|  | 3.7 Other data sources |  |
| 1. Cross-sectoral Resource Mobilization and Allocation | 4.1 Prioritization of resources | 4.1.1 Sustainability of resources |
|  | 4.2 Alignment of Sectoral Priorities | 4.2.1 Joint Implementation of sectoral priorities |
|  | 4.3 Pooled Funds | 4.3.1 Mechanism for Pooling Funds |
|  |  | 4.3.2 Access to Pooled Funds |
| 1. Stakeholder Engagement and Joint Decision making | 5.1 Communities | 5.1.1 Involvement in JRA process |
|  |  | 5.1.2 Involvement in identification of Risk Mitigation interventions |
|  |  | 5.1.3 Level of Risk Perception |
|  | 5.2 Decision Makers | 5.2.1 Awareness about One Health |
|  |  | 5.2.2 Prioritization of JRA process |
|  | 5.3 Other relevant sectors | 5.3.1 Representation in JRA/Integrated Risk Analysis processes |
|  | 5.4 Joint Decision making | 5.4.1 Consensus Building Process |
|  |  | 5.4.2 Alignment of Sectoral Priorities |
|  |  | 5.4.3 Joint Risk-based Decision making |
|  |  | 5.4.4 Data-Driven Decision making |
| 1. Cross-sectoral Risk Mitigation and Communication | 6.1 Preparedness Interventions |  |
|  | 6.2 Surveillance and Early Warning Systems | 6.2.1 Disease-specific |
|  |  | 6.2.2 All-Hazards |
|  |  | 6.2.3 Pathogen-Agnostics Testing |
|  |  | 6.2.4 Systems Interoperability |
|  | 6.3 Joint Surveillance and Reporting |  |
|  | 6.4 Joint response |  |
|  | 6.5 Health Systems resilience building | 6.5.1 Adaptability to environmental/climate hazards |
|  |  | 6.5.2 Recovery from Public Health threats/shocks |
|  | 6.6 Community engagement and sensitization |  |
|  | 6.7 Cross-sectoral information and knowledge management | 6.7.1 Knowledge generation |
|  |  | 6.7.2 Knowledge dissemination |
|  | 6.8 Workforce Capacity strengthening | 6.8.1 Capacities for Joint Rapid Response |
|  | 6.9 Cross-sectoral Operational Readiness | 6.9.1 Activation of Joint Rapid Response Teams |
|  |  | 6.9.2 Simulation exercises |
| 1. Enhancing Coping capacities for Pandemic Prevention and Risk Reduction | 7.1 Integration of Environmental strategies and policies into OH approach | 7.1.1 Mechanisms of integration |
|  |  | 7.1.2 Structures for Integration |
|  | 7.2 Climate Change and Environment Policies |  |
|  | 7.3 Institutional Capacities | 7.3.1 Cross-sectoral polices and action plans |
|  |  | 7.3.2 Governance |
|  |  | 7.3.3 Disaster Risk Reduction capacities |
|  | 7.4 Infrastructure capacities | 7.4.1 Level of access to Climate-resilient Health systems |
|  |  | 7.4.2 IHR Implementation Capacities |
|  |  | 7.4.3 ICT |
