# Supplementary material for: Improving risk analysis of environmentally driven zoonotic biological threats as a primary pandemic prevention approach: A case study of the Tripartite Joint Risk Assessment Operational Tool operationalization in Kenya
Source: PLOS Glob Public Health. 2026 Jul 1;6(7):e0006560. doi: 10.1371/journal.pgph.0006560 (PMC13322548; doi:10.1371/journal.pgph.0006560)
Supplement: S1 Appendix — (DOCX) [file pgph.0006560.s004.docx]

**S1 Appendix. Findings from a Stakeholder Validation workshop scenario planning exercise on addressing integrated data gaps.**

Table A: Context and Risk Scenarios using the three dimensions of risks under the IRA framework (adapted from the INFORM Global Risk/Epidemic Risk Index)

| Context/Risk Scenario | Hazards and Exposures | Population Vulnerabilities | Lack of Coping Capacities |
| --- | --- | --- | --- |
| 1. Increase in flooding events in Tana River County following El-Nino rains/ risk of severe outbreak of Crimean-Congo Hemorrhagic Fever (CCHF) | - Extreme weather events - Increase abundance and survivability of CCHFV-infected tick population | Loss of livelihoods due to extreme weather events increases human displacement and population migration; humans live closer to wildlife | Poor infection prevention control and disease prevention practices; limited capacities at points of entry |
| 1. Increasing uptake of fruit farming in Bungoma county/ risk of Marburg Virus Disease (MVD) emergence in non-endemic areas | Increase in activities that facilitate human exposure to MARV-infected fruit bats and MVD outbreaks. | Migration of MARV-infected bats to new roosting sites increases risk of emergence of new viral strains to non-endemic areas | Limited diagnostics capacity serves as impediment to early detection |
| 1. Increased reliance on camel-rearing as a form of drought resilience in Marsabit county; cultural attachment to animals/risk of Middle Eastern Respiratory syndrome (MERS) outbreak with sustained human-human disease transmission | Increasing frequency of climate change-induced drought events; increasing density of camels in the region with evidence of exposure to MERS-CoV | Livestock loss due to droughts results in increased population demand for camel rearing. | Lack of Data; limited knowledge about population susceptibility |

Table B: Presentation of Results from Group Work Discussions

| Guiding Questions for Group Discussion | **Group A: Tana River County/ CCHF** | **Group B: Bungoma County/MVD** | **Group C: Marsabit County/MERS** |
| --- | --- | --- | --- |
| 1. What sectors are important to communicate with to obtain required data for integrated risk analysis of given hazards and exposure? | Areas of Expertise: Clinicians, Public Health Officers, Laboratory Services, Veterinarians,  Entomologist.  Sectors: Ministry of Environment, Climate Change and Forestry; Kenya Meteorological Department; Kenya Wildlife Services; Ministry of Health, Department of Disease Surveillance and Response; Ministry of Agriculture, Livestock and Fisheries; National Migration Coordination Mechanism, Department of Immigration Services | Ministry of Health; Ministry of Agriculture, Livestock, and Fisheries; Kenya Wildlife Services; Ministry of Environment, Climate Change and Forestry, Kenya Meteorological Department | Human Health, Animal Health, Environment, Trade, Research Institutions, Members of the Quadripartite Alliance |
| 1. What other relevant data is needed to carry out the EID risk assessment? | Encroachment of wildlife areas, Migration patterns | Seasonality, Food security data, Location of fruit traders, surveillance data | Population Density (Camels and Humans), Weather variability data, Epidemiological data, Risk profile, Human syndromic data, Animal syndromic data, Meteorological data |
| 1. How might data sharing arrangements look like under the given scenarios? | Bottom-up data sharing arrangements from communities to counties; counties to national; national to regional; and regional to international | Multisectoral data sharing One Health platforms for data sharing | Alternative data sharing arrangements like email listservs, and whatsapp group data sharing |
| 1. What policies exist/are required to enable cross-sectoral data sharing? | Revisions can be made to existing policies to enable data sharing including Public Health Act, Kenya Veterinary Policy, Kenya Climate Change act (Amendment 2023) | No policies exist, One Health policies are required | No policies exist. The recommendation is to establish an MOU that outlines: host institution responsible for data; associated costs; types of data (indicators) to be shared; level of data sharing |
| 1. What types of platforms are available or needed for data integration and interoperability? | Kenya Health Information System, Kobo Toolbox | Kenya Health Information System, Event-based Surveillance Systems, Integrate relevant data from Central Bureau of Statistics, Kenya Animal Biosurveillance Systems | Kenya Environmental Management System, Kenya Health Information Systems, Kenya Animal Biosurveillance System |

Table C: Final List of Presented Variables by Category

| Variable Category | **CCHFV** | **MARV** | **MERS-CoV** |
| --- | --- | --- | --- |
| Epidemiological/ Syndromic | Incidence and Prevalence | Incidence and Prevalence | - Incidence - Seroprevalence - Animal Syndromic data - Human Syndromic Data |
| Environmental/  Socioanthropgenic | - Average Annual Change in Nighttime Light Index - Land cover - Non-irrigated agricultural land cover (for e.g. pasture and rangeland) - Cattle Density - Shrub Type - Enhanced   Vegetation  Index   - Normalized Difference Vegetation Index - Percent Sand-soil content - Encroachment areas | - Enhanced Vegetation Level - Vegetation Level - Forested land fragmented by agriculture or shrub cover | - Population Density (Human and Camels) - Land Cover - Bare land coverage - Forest Coverage - Dry season grazing area |
| Climatological | - Land surface temperature (mean temperature of wettest and driest time of the year) - Precipitation (of wettest quarter of the year) | - Temperature Seasonality - Rainfall (mean annual, driest quarter) | - Annual Mean temperature - Frequency of drought events |
| Socioeconomic | Migration Patterns | - Food security - Location of fruit traders | Number of camels per households |
