# Supplementary material for: Improving risk analysis of environmentally driven zoonotic biological threats as a primary pandemic prevention approach: A case study of the Tripartite Joint Risk Assessment Operational Tool operationalization in Kenya
Source: PLOS Glob Public Health. 2026 Jul 1;6(7):e0006560. doi: 10.1371/journal.pgph.0006560 (PMC13322548; doi:10.1371/journal.pgph.0006560)
Supplement: Attachment — Submitted filename: Updated response to peer reviewer_042026.pdf [file pgph.0006560.s006.pdf]

April 11, 2026

Oluwayemisi Ajumobi
Johns Hopkins University
615 N Wolfe Street
Baltimore, MD 21205, USA
oajumob1@jhu.edu

Dr. Catherine Kyobutungi and Dr. Madhukar Pai
Editors-in-Chief
PLOS Global Public Health
1875 Mission Street, Suite 103 #188
San Francisco, CA 94103

Dear Drs., Kyobutungi and Pai,

Re: Point by Point Response to Additional Feedback from Peer Reviewer on Qualitative Study manuscript (ID: PGPH-D-25-00514) submitted for publication in PLOS Global Public Health Journal

On behalf of the research study team, thank you for providing the opportunity for peer reviewers to share their feedback on the qualitative study manuscript (**ID: PGPH-D-25-00514**) titled **"Improving risk analysis of environmentally driven zoonotic biological threats as a primary pandemic prevention approach: A case study of the Tripartite Joint Risk Assessment Operational Tool operationalization in Kenya"**. Many thanks for the additional comment provided by Peer Reviewer 1. In response to the comments from the reviewer, the following points below were addressed in the revised manuscript:

Peer Reviewer 1

Thank you for the explanations provided in your response to editors and reviewers comments. This helped to clarify the objectives and outcomes of the research and the timing/positionality of IRA and JRA enormously. However, with the greater understanding you offered in your responses, I am afraid that more serious concerns emerge emphasizing that the current presentation of your framework was not – and remains not - completely clear.

- The abstract refers to the IRA but gives no indication where this framework comes from and where it in in time in relation to this paper – does it already exist? Is this research informing its development? Are you presenting your framework here, as figure 1? If it is figure 1 (although it seems to only be a component of figure 1), the paper does not seem to actually present it and the data obtained in this study would anyway be insufficient for developing an entire framework.
  Authors' response: Thank you for the comment. The IRA is a new framework conceptualized from a previous study by Ajumobi et al. (2025). This framework is discussed in the introduction section (page 5) and referenced in the methods section (pages 8 and 9) and other subsequent sections of this study including the discussion section (page 20). As mentioned in the abstract, our aim was to obtain the perspective of stakeholders on the

development of this newly conceptualized IRA framework. In page 5, we mention that to address this study aim, we conducted key informant interviews "to learn from the experiences of stakeholders with the operationalization of the JRA OT in Kenya and to obtain their inputs on essential components required to improve environmentally driven biological threat risk analysis processes at the environment-animal-human exposure interface. Information obtained from the study was used to finalize the development of an integrated risk analysis (IRA) framework for pathogen/disease spillover, emergence/reemergence and spread (SES) risk analysis to inform cross-sectoral pandemic prevention decision making.' In figure 1, we have presented our findings from this research as the essential component of the IRA framework that has evolved from the initial conceptual framework from the prior study to the framework presented as figure 1 informed by finding from our study. To address this comment, we have revised the background section of the abstract to include a reference to the newly conceptualized framework as follows: "We aimed to obtain the perspectives of stakeholders on the development of <u>a newly conceptualized</u> integrated risk analysis (IRA) framework to address environmentally driven EIDs."

- Similarly, the cover letter suggests the newly developed IRA will be presented, and the paper refers to the development of such a a newly conceptualized integrated framework for risk analyses but the abstract states 'Improving IRA processes… will…' suggesting that IRA processes already exist, but also states that 'Information obtained from the study was used to finalize the development of an integrated risk analysis (IRA) framework for pathogen/disease spillover, emergence/reemergence and spread (SES) risk analysis to inform cross-sectoral pandemic prevention decision making' yet, again, the framework seems not to actually be presented/described in the paper and there is no mention of where that framework might be found. All this, including where the framework is, must be explained and clarified in the abstract and early in the paper.
  Authors' response: Thank you for the comment. The newly conceptualized IRA framework is mentioned in the introduction section and described in the methods and discussions sections of the manuscript. To address this comment, we have also included a reference to the associated study on the conceptualization of the IRA framework in the last paragraph of the introduction section of the manuscript (page 5).

- The title should be changed to highlight that this is a qualitative study to learn mostly about integrating environment in RA, based partially on application of the JRA IN KENYA, not a case study or evaluation of the JRA tool itself (as per response to reviewer 2) and certainly not its operationalization. The cover letter also suggests Kenya will be used as a case study, which is truer than suggesting that the JRA will be used as one. The abstract – which I think describes the study quite accurately, only peripherally refers to the JRA.
  Authors' response: Thank you for the comment and suggestion. We agree that the study is not an evaluation of the JRA process. The manuscript title mentions that the study is a qualitative study. Our study was primarily informed by insights and lessons from the JRA operationalization in Kenya which served as a case study to learn about the processes (including best practices and challenges) of operationalizing the OH approach in Kenya with a specific focus on the JRA operationalization process. For example, participants shared

experiences and lessons learned from the operationalization of the JRA OT in four Western counties included in the study (Busia, Bungoma, Kakamega, and Vihiga) to jointly assess priority zoonotic diseases and risk of introduction of Ebola Virus Disease (EVD) into Kenya from Uganda following the start of the EVD outbreak in September 2022 Hence, we consider it appropriate to highlight "a case study of the Tripartite Joint Risk Assessment Operational Tool in Kenya" in the title to better reflect the study context. To address this comment, we have included important aspects of our study findings in relation to the JRA OT operationalization in the discussion section (pages 20-21). Also, we have added a sentence in the background section of the abstract as follows: "We addressed the study aim by learning from stakeholders' experiences with the operationalization of the Joint Risk Assessment Operational Tool (JRA OT) in Kenya."

- *Please correct WOOAH*
  Authors' response: Thank you for the correction. We have changed WOOAH to WOAH in page 5 of the manuscript.

- Is the term conceptual framework referring to the IRA framework? Please clarify use of the terms.
  Authors' response: Thank you for the comment. The term conceptual framework refers to the newly conceptualized IRA framework from the study by Ajumobi et al (2025). To address this comment, we have clarified the term usage in the first sentence under the data collection procedure sub-section (page 8) as follows: "For data collection, we developed an interview guide using key elements of the initially developed IRA conceptual framework described in a related study by Ajumobi et al (2025).[20]"

- Please comment/clarify in the document the extent of difference or overlap of i – iv concepts with basic principles of the JRA OT itself
  Authors' response: Thank you for the valuable suggestion. We have included both the distinctions and similarities between the JRA OT and the IRA framework in the discussion section of the manuscript (pages 202-21), as follows: "Important insights emerged from our qualitative case study about the JRA operationalization process in Kenya. A key distinction between the JRA process, a curriculum-based, 10-step process focused on the process of conducting joint risk-based activities across sectors, and the IRA framework described in Figure 1 is in the use of the JRA OT for addressing specific health threats including cross-border outbreaks. In 2022, for example, the JRA OT was operationalized in four Western counties included in the study (Busia, Bungoma, Kakamega, and Vihiga) to jointly assess priority zoonotic diseases and risk of introduction of Ebola Virus Disease (EVD) into Kenya from Uganda following the start of the EVD outbreak in September 2022.28
  Like the JRA process, the IRA framework model promotes the involvement of different stakeholders across relevant sectors and goes a step further by highlighting the importance of holistically engaging communities as key stakeholders in IRA and JRA processes. However, in contrast to the JRA process, the IRA framework model utilizes a multihazards

scenario approach that incorporates environmental parameters into risk models. As a data-driven risk-based framework that enables the use of both quantitative and qualitative data in the risk models, the IRA framework adopts environmental monitoring techniques and systematically accounts for risk-based measures across the entire spectrum of risk analyses processes. These measures include eco-epidemiological, socio-economic and other cascading risk factors that facilitate the spillover, emergence/reemergence, and spread (SES) of EIDs as an essential component of IRA in relation to the need for improving cross-sectoral data sharing and integration. Inclusion of these variable types under the IRA model is critical for informing data-informed risk mitigation and communication interventions, proactive resource mobilization, and implementation of future environmentally driven spillover and outbreak prevention strategies.

Another distinction of the IRA framework from the JRA OT is the inclusion of an evaluation component that describes essential factors to consider in evaluating the level of enhanced coping capacities due to IRA processes. Addition of this component under the IRA framework aligns with policy recommendations for improving OH risk governance and regulations. Overall, the IRA framework model utilizes both a cross-sectoral and multihazards scenario approach that integrates risk modeling data (in addition to surveillance data and qualitative data) and environmental parameters into risk models. Based on the insights that emerged from our study, we consider findings from the JRA exercises to be a complementary source of qualitative data under the IRA framework model."

- Also, although you made some smaller changes and clarified several more complex points for the editor and reviewer in your responses, your comments did not translate into edits allowing better understanding for the reader. These, and a reflexive review of the above concerns, should allow development of a clearer presentation within the paper of these issues.

  Authors' response: Thank you for the comment. In addition to the changes made to earlier version of the manuscript, we have taken this comment into account by including additional changes to the manuscript to reflect both changes made in response to prior comments, where applicable, and to the additional feedback received.

Thank you, once again, for further considering our manuscript for publication in your journal. I hope you find the points addressed in the resubmitted version of the manuscript to your satisfaction. We look forward to hearing from you about the outcome of our submission.

Sincerely,

Oluwayemisi Ajumobi
