# Supplementary material for: Improving risk analysis of environmentally driven zoonotic biological threats as a primary pandemic prevention approach: A case study of the Tripartite Joint Risk Assessment Operational Tool operationalization in Kenya
Source: PLOS Glob Public Health. 2026 Jul 1;6(7):e0006560. doi: 10.1371/journal.pgph.0006560 (PMC13322548; doi:10.1371/journal.pgph.0006560)
Supplement: Attachment — Submitted filename: Response to reveiwers.docx [file pgph.0006560.s005.docx]

Oluwayemisi Ajumobi

Johns Hopkins University

615 N Wolfe Street

Baltimore, MD 21205, USA

[oajumob1@jhu.edu](mailto:oajumob1@jhu.edu)

Dr. Catherine Kyobutungi and Dr. Madhukar Pai

Editors-in-Chief

PLOS Global Public Health

1875 Mission Street, Suite 103 #188

San Francisco, CA 94103

Dear Drs., Kyobutungi and Pai,

Re: Point by Point Response to Feedback from Peer Reviewers on Qualitative Study manuscript (ID: PGPH-D-25-00514) submitted for publication in PLOS Global Public Health Journal

On behalf of the research study team, thank you for providing the opportunity for peer reviewers to share their feedback on the qualitative study manuscript (**ID: PGPH-D-25-00514**) titled **“Improving risk analysis of environmentally driven zoonotic biological threats as a primary pandemic prevention approach: A case study of the Tripartite Joint Risk Assessment Operational Tool operationalization in Kenya*”****”*. Many thanks for the editorial comments and to the two reviewers for taking out time to review and provide constructive feedback on the manuscript. In response to the editor and reviewers’ questions and feedback, the following points were addressed in the resubmitted manuscript below:

**Additional Editor Comments**

1) Please refer to comments from both the reviewers on the aims and design of your study, including the additional value from an IRA.

*Authors’ response: Thanks for the comment. We have addressed these comments in corresponding responses to the reviewers. The added value of an IRA framework is in the use of the framework for the integration of environmental monitoring techniques under risk analysis processes. This is a major gap in existing tools including the JRA. To emphasize, the IRA framework is a data-driven One Health risk-based framework that enables the integration of environmental monitoring techniques across the entire spectrum of risk analyses processes (beyond risk assessments), and the systematic inclusion of eco-epidemiological, socio-economic and other cascading risk factors that facilitate the spillover, emergence, and spread of EIDS; application of these types of variables under the IRA model is critical for informing data-driven risk mitigation and communication interventions (including data-informed decision making and resource allocation), and future environmentally driven spillover and outbreak prevention strategies. While the JRA process is carried out for specific biological threat (with the joint aspect referring to involvement of cross-sectoral stakeholders in the process), the IRA framework model utilizes a multihazards scenario approach that incorporates environmental parameters into risk models, in addition to the involvement of different stakeholders across relevant sectors.*

*Another added value of the IRA framework is the utility of the framework for enhancing the operationalization of the One Health approach and the inclusion of an evaluation component that describes essential factors to consider in evaluating the level of enhanced coping capacities. Addition of this component under the IRA framework accordance with policy recommendations for improving OH risk governance and regulations.*

2) It is not clear to me how you understand environment health / hazards and also what might 'environment sector' mean in the context of your study site (Kenya) as well as more widely in other countries. It will be helpful if you could define these concepts and clarify your stance.

*Authors’ response: Thanks for the constructive feedback. We have provided a clarification of these concepts in page 4 of the revised manuscript, as follows: “Within the study context, in line with the UNEP Strategy for Environmental Education and Training, the environment sector refers to industries and activities encompassing the natural and built environment, sociological and economic aspects of environmental issues, and the political dimensions of environmental protection and resource management. Further, as classified under the Index for Risk Management Global Risk Index (INFORM GRI), environmental hazards include floods, droughts, earthquakes, tsunami, tropical cyclones, and epidemics.”*

**Peer Reviewer 1**

- This paper provides important information on application of the JRA tool from users in Kenya. The key themes that emerged were very interesting and operationally important, but none are excluded within the existing JRA process. The relationship of the proposed IRA and the existing JRA OT (as well as the other RA frameworks mentioned) is unclear, particularly the specifics of the proposed IRA and how the proposed IRA differs in operational substance from the JRA.

  Similarly, it is unclear why improved application of the existing JRA tool by inclusion / integration of information and actors from the environment sector, rather than creating a new framework with a new name, would be required to account for e.g. environmental drivers and why the other aspects of the output triad could not fit in the existing JRA process.

  The main added value seems to be a recommendation of ensuring the JRA risk management and communication outcomes are implemented in policy including environmental impact assessments, but no specific operational solution is provided. Would seem to be more useful to present the information as specific guidance to improve scope and sustainability of outcomes for those using the JRA and practical ways of working outcomes into policy.

*Authors’ response: Thanks for the comment. The added value of the IRA framework, a data-driven (quantitative and qualitative) risk-based framework model are as follows:*

- *Application of environmental monitoring techniques and accounting for risk-based measures across the entire spectrum of risk analyses processes i.e. beyond risk assessments (the focus of the JRA as a qualitative structured curriculum), and the systematic inclusion of eco-epidemiological, socio-economic and other cascading risk factors that facilitate the spillover, emergence, and spread of EIDs. Inclusion of these variable types under the IRA model is critical for informing data-informed risk mitigation and communication interventions, and future environmentally driven spillover and outbreak prevention strategies. While the JRA process is typically carried out for specific biological threat (with the joint aspect largely referring to cross-sectoral involvement in the process), the IRA framework model utilizes both a cross-sectoral and multihazards scenario approach that integates risk modeling data (in addition to surveillance data and qualitative data) and environmental parameters into risk models*
- *Enhances the operationalization of the One Health approach by accounting for the core IRA operationalization triad across every step of the risk analysis process (beyond risk assessments)*
- *The inclusion of an evaluation component under the framework is another important distinction. This component of the IRA accounts for essential factors to consider in evaluating the level of enhanced coping capacities attributable to IRA processes and aligns with policy recommendations for improving OH risk governance and regulations.*

*Further, as highlighted in the discussion section, findings from this study complement prior work from a related study on identifying the most relevant environmental monitoring, climatological, and socioecological variables that could be used to improve risk characterization and subsequent stages of risk analysis – an aspect the IRA model thoroughly captures. Our study findings also informs a subsequent interconnected policy paper that directly addresses the comment about operational solutions for improving risk analysis processes. To summarize the distinction between the JR and IRA, it’s also important for us to emphasize that findings from JRA exercises can serve as good sources of qualitative data for the use of the IRA framework model (which is not intended to be a substitute to the JRA).*

- Summary: The environmental aspects highlighted were not included specifically as essential component outcomes of the interviews. Need to link/align results with analysis.

*Author response: Thanks for the comments The environmental aspects highlighted relate to the listed components under the operationalization triad on cross-sectoral data sharing and integration including the need for better data derived from environmental monitoring, data sharing and reporting arrangements, cross-sectoral risk mapping, technologies, and environmental policies. The environmental risk factors that emerged from tthe study did not emerge as essential components of an operational framework but were considered as minor themes all relevant to the included operationalization triad on cross-sectoral data sharing and integration.*

- P4 ‘The JRA OT is one of three operational tools … to qualitatively assess risks posed by zoonotic disease hazards at the human-animal-environment interfaces, and at the national and sub-national levels.’ – there are not three RA OT tools, but three tools overall.

*Authors’ response: Thanks for the comment. We agree with the distinction provided. In our introduction of the origins of the JRA OT, we highlighted that the JRA OT is one of three* ***operational tools*** *under the Tripartite zoonosis guide (and not risk assessment OT). These operational tools include the JRA OT, the Multisectoral One Health Coordination Mechanism (MCM OT), and the Surveillance and Information Sharing Operational Tool (SIS OT).*

- Describe any efforts at improving the gender balance as well as the balance across sectors and disciplines and specifically how that could have impacted the results.

Authors’ response: Thanks for the comment. We addressed this comment in the methods section of the manuscript as follows: *“Potential participants were identified and nominated by the ZDU from a list of attendees of past JRA OT training workshops and other related OH trainings. We utilized a stratified purposeful and maximum variation sampling technique for the nomination of participants from a larger list to ensure ample diversity in representation of participants at different level of engagements (national- and county-level governments, and partner institutions) and across different counties and sectors. Snowballing was also used to identify additional participants.”*

*In the limitations section, we also acknowledged the limited number of female participants, as follows: It is worth noting that the limited number of nominated female participants who met the study selection criteria shed light on a broader issue regarding the need for a balanced gender representation in the operationalization of the OH approach to better address gender-related vulnerabilities to infectious diseases. However, the study team acknowledges that the observations around the gender imbalance is beyond the scope of this study and could help inform future efforts.*

- Quality of fig 1 could be improved, including what aspects are included in the JRA and the other RA frameworks mentioned.

*Authors’ response: Thanks for the observations. We have revised figure 1 to make the figure more visually accessible. As provided in the manuscript, conceptualization of the IRA framework was adapted from the INFORM GRI and WHO Health EDRM. The study team learned from the process of conducting joint risk assessments across sectors. However, the team recognizes that the JRA, a curriculum-based, ten steps process is focused on the process of conducting a joint risk-based activities across sectors, and is distinct from tthe IRA framework described in figure 1,*

**Peer Reviewer 2**

- Congratulations to the authors of this study! In my opinion, this study aimed to evaluate the usefulness of the Joint Risk Assessment (JRA) Operational Tool, focusing on conducting integrated risk assessments of infectious disease risk caused by environmental changes. I find the topic highly relevant because, to harvest the benefits of the One Health approach, all stakeholders must actively participate in and contribute to One Health initiatives. In my experience, bringing the human and animal health sectors together has been a challenge, but not as challenging as getting the environmental sector on board. The challenge is reflected in the late addition of UNEP to the institutions supporting the JRA tool. I applaud the authors' efforts to bring the environmental perspective into the debate. I believe the timing is opportune because, as the authors mention, the increase in extreme weather events may increase the threat of emerging or re-emerging infectious diseases. In my opinion, the impact of this study is the development of a new integrated risk analysis (IRA) framework that better incorporates the environmental sector, in addition to addressing other areas that the study identifies as areas for improvement.

*Authors’ response: Thank you for the comments. We agree with your comment including the observations about the study’s impact by both ensuring better integration of the environment sector into the OH approach and enhancing the operationalization of OH and governance mechanisms.*

Major issues

 I suggest that the authors review the aim of their study. In my opinion, the aim is to evaluate the usefulness of the JRA-OT or the usefulness of the current IRA framework. This is because:
a. The participants were required to be acquainted with the JRA-OT, and many of the questions were about the JRA-OT, not other frameworks.
b. The abstract states that the aim is to “obtain the perspectives of stakeholders on the development…” However, the participants were not asked for their perspectives on a new proposed IRA framework, but rather their experiences with the current framework.

*Authors’ response: Thank you for the comments. The main rationale for focusing on the JRA OT was to learn from the process of bringing multiple sectors together because we believe these lessons are directly transferrable to other integrated cross-sectoral risk-based approaches focused on enhancing the operationalization of the OH approach. Despite the focus on the JRA OT as a case study, our goal was not to evaluate the JRA OT, but to learn from an existing systematic process of bringing sectors together (a process that the JRA OT was created to perform notwithstanding existing challenges with the inclusion of the environment sector in the process). On the other hand, the newly developed IRA framework is a data-driven framework model (utilizing both quantitative and qualitative data sources including stakeholder opinions) that accounts for the utilization of data and incorporation of other aspects of the core IRA operationalization triad across the entire spectrum of risk analysis (risk assessments, risk characterization, risk mitigation, and risk communication), to inform risk-based decision making and resource allocation.*

*Our proposed IRA model also incorporates a critical data-driven evaluative component in the early stages (and not as a post-implementation impact evaluation activity) as a strategy to improve OH governance. While we utilized the JRA OT as a case study to learn about the processes (and challenges) of operationalizing the OH approach in Kenya, the study team adopted a good balance of both JRA-specific questions and non-JRA specific questions in the semi-structured interview guide. Some questions directly asked participants to provide their perspectives on essential components related to IRA/non-JRA specific processes (please refer to the section on understanding the environment-infectious disease interface****: for e.g. question*** ***viii) In your opinion, what are essential risk components that must be accounted for to ensure an early and effective response to environmentally driven EIDs at the source?***

c. The four major themes that emerged from the study are not limited to risk analysis for infectious diseases driven by environmental changes. The improved IRA will not only address the inclusion of the environmental sector, but also these four themes.

*Authors’ response: Thanks for the comment. We agree that the utility of the newly developed IRA framework goes beyond inclusion of the environment sector but would help enhance operationalization of a risk-based OH approach at the animal-human-environment interface; and the overall governance of OH at national and sub-national (county) levels. This was an important overarching finding of the study and a major outcome of the stakeholder validation =n workshop..*

Minor issues

- Abstract: The abbreviation “JRA” is not defined in the abstract. Add what JRA stands for in the abstract.

*Authors’ response: Thanks for the helpful observation. In the interpretation section of the abstract, we have spelled out the full acronym for JRAs as follows: Joint Risk Assessments (JRAs).*

- Introduction: To the sentence “Hence, in addition to priority endemic zoonotic diseases (such as anthrax, brucellosis, bovine tuberculosis, and rabies)” provide a reference. Who established these diseases as priorities?

Authors response: Thanks for the comment. In the introduction section, we provided a reference to the Kenya National One Health Strategic Plan (2021-2015). To address the comment about the reference source, we made minor revisions to the sentence in the manuscript, as follows: *Hence, in addition to priority endemic zoonotic diseases (such as anthrax, brucellosis, bovine tuberculosis, and rabies) listed under the 2021-2025 National One Health Strategic Plan.(8), EIDs are also a major concern to the Government of Kenya and are prioritized in the strategic plan.*

- Introduction: Are the pathogens of focus, CCHFV, MARV, and MERS-CoV, environmentally driven? According to the introduction, only MARV is environmentally driven.

Authors response: Thanks for the question. All three pathogens of focus are classified as environmentally driven. We have expounded on this point with references provided ss follows: *The rationale for the selection of these three pathogens include increasing evidence of CCHFV and MERS-CoV seroprevalence across Kenya linked to climate change-driven changes in animal, human and wildlife population migration patterns, and risk of environmentally driven MARV spillover events and potential to sustain human-to-human disease transmission.(9–12)*

- Introduction: Describe what the studies 13-15 claimed. Explain what the evidence or lack of evidence exists to call for “a better understanding and synthesis of probable environmentally driven disease transmission risk pathways”.

*Authors’ response: Thanks for the comment. We have accounted for this comment in the revised manuscript, as follows: The study by Loh et al. (2015) explored the major environmental drivers of EIDs of zoonotic origins and highlighted the importance of targeting surveillance and control activities to address different transmission pathways. To make the case for a better understanding of the eco-social processes that facilitate disease emergence and spread under risk analysis. Jones et al (2016) provided examples of emergence and spread of different pathogens including previously unknown pathogens like Nipah virus, MERS-CoV, and Avian Influenza; and pathogens with extended geographic range such as West Nile virus, and Ebola. Similarly, Sorvillo et al. (2020) discussed the need for joint risk assessments of zoonotic biological threats like CCHFV by utilizing a comprehensive framework that combines epidemiological, ecological, virology and vector biology data, with modeling data to make an estimation of outbreak risk and target surveillance activities*

- Methods: In the description of the eligibility criteria, clarify whether all criteria were required (AND) or only one (OR).

*Authors’ response: Thanks for the suggestion. We have provided clarification about the eligibility criteria in the methods section, as follows: All eligibility criteria were required to participate in the study.*

- Methods: What were the categories (and the weight of each category) planned for the stratified sampling?

*Authors’ response: The categories utilized for the stratified sampling were primarily by stakeholder group (national level government/county level government/ partner institutions) and sectoral representation (human health, animal/veterinary health, environment, and wildlife). Equal weights were assigned across categories by stakeholder groups in the initial nomination of participants.*

- Methods: I suggest moving the number of declinations to participate to the results section. Describe the characteristics of those who decline. Were they appointed to the government or the quadripartite institutions?

*Authors’ response: Thanks for the suggestion. As described in the study manuscript, we utilized the consolidated criteria for reporting qualitative research (COREQ) checklist to report the information provided in the manuscript. The guidance provided under the COREQ checklist is to report the number of non-participation in the methods section. The only participant that declined participation in the study was from a partner institution. Other nominees unavailable to participate in the study (due to travel schedule and transfer to a different county) were representatives from partner institutions and government agencies respectively. We have revised the applicable section in the manuscript to account for this comment, as follows: For the final study sample, one participant from a partner institution declined, three participants representing a partner institution and government agencies were unavailable due to scheduling conflicts (n=2) and a transfer to a different county (n=1),*

- Methods: Some interviews were conducted at work. How was privacy safeguarded? Is it possible that participants felt obliged to respond in a certain way to please the view of their institution?

*Authors’ response: Thank you for this important comment. The interviews conducted at the work locations of participants took place in private offices selected by participants (with only the interviewer and, in some cases, a research assistant present in the office with the participant. As a general observation, all participants provided an open, unscripted response throughout the interview sessions and did not appear to be under pressure to respond to questions in favor of their institutions. Although we utilized a semi-structured interview guide*, the interview sessions followed a natural progression.

- Methods: I suggest not referencing a framework located in the protocol unless the protocol can be accessed.

*Authors’ response: Thank you for the suggestion. We have provided a reference to another related study where the framework is described. Additionally, we modified the sentence in the manuscript to read, as follows: For data collection, we developed an interview guide using key elements of the conceptual framework described in a related study by Ajumobi et al (2025).*

- Methods: Clarify how INFORM GRI and Health EDRM were used.

*Authors’ response: The INFORM GRI and Health EDRM were considered the most appropriate for developing the conceptual IRA framework as they both utilize a mulithazards risk-based approach..We adopted key elements of the two frameworks including the three dimensions of risk under the INFORM GRI (derived as Hazards and exposures X population vulnerabilities X lack of coping capacities) for assessing risk levels and for the risk characterization. We also combined components of the WHO Health EDRM which adopts an all hazards approach to disaster risk management for risk mitigate and communication. These components were included in the conceptual framework used to develop the study interview guide, as follows: 1) risk based all-hazards surveillance and early warning systems; 2) cross-sectoral information and knowledge management; 3) community engagement; 4) health workforce capacity strengthening; and 5) operational readiness and simulation exercises*

- Methods: The description of Table 1 in the methods may not correspond to Table 1 in Results.

*Authors’ response: Thanks for the observation. We acknowledge that the initial categorization of finance as a standalone sector under Table 1 may cause a discrepancy in the interpretation of the results table which should be interpreted in parallel to the information provided in the methods section. However, the perspective obtained on finance was from a participant who also represented the animal health/veterinary sector. To address this comment, we have revised Table 1 to integrate the data of the participant representing both finance and veterinary health (n = 1) under the listed animal/veterinary sector. We also maintained the need to recognize the perspective obtained from the same participant on resource mobilization, listed as an area of specialty under the same table.*

- Results: Describe the 14 participants included in the validation workshop. Comment about the representativeness of the sample to the interviewed population.

*Authors’ response: The 14 workshop participants were selected from the list of study participants. The criteria used for their selection was to ensure all counties, sectors, and partner institutions were represented, and a relatively balanced representation of participants across sectors and stakeholder categories.*

- Discussion: Please clarify how this study aligns with “data-driven risk analysis-based strategies”. The JAR-OT is a qualitative tool that could rely on expert opinion.

*Authors’ response: Thanks for the remark. We agree with the comment that the JRA OT is a qualitative tool that relies on expert opinion. The statement that our “study findings align with recommendations for adopting data driven risk-analysis-based strategies…” directly confirms the need for utilizing a data-driven IRA model (a model that utilizes a combination of surveillance data, predictive risk models and qualitative expert opinions) to fill data gaps in joint assessment process like the JRAs – a prominent theme that emerged from our study*

- Discussion: Consider comparing the usefulness of INFORM GRI, Health EDRM, and JAR, focusing on the environmental component of OH.

*Authors’ response: Thank you for the suggestion. We see the value of conducting this exercise, However, we consider this exercise to be more appropriate for a different study that seeks to address a question on the utility of these tools in relation to the environmental component of One Health.*

- Discussion: What policies are currently in place in Kenya? In the discussion, the authors indicate the “need for the development and implementation of OH policies.” What should be included in these policies?

*Authors’ response: Thank you for the questions. Based on our study findings, there were no existing OH policies in Kenya. At the time the study was conducted, some participants shared that the process of developing and enacting a OH policy was underway. In reference to the specific sentence included in the manuscript, we discussed the need to incorporate environmental sectoral priorities into OH policies. An example of strategies to be included in these policies is provided in the discussion section of the manuscript, as follows: A feasible OH policy option that emerged from our findings is to incorporate the risk analysis of environmental drivers of zoonotic biological threats (in relation to SES) as a mandatory part of environment impact assessments. This approach to IRA may serve as a mechanism for addressing shared commonalities across sectors, in line with similar recommendations from previous assessment studies.(28,29)*

- Supplement: What are the main differences between versions 1 and 2 of the semi-structured interview guides?

*Authors’ response: Thanks for the thoughtful question. The table below provides a breakdown of the main differences between the two versions of the semi-structured interview guide*

| ***Semi-structured interview guide (Version 1)*** | ***Semi-structured interview guide (Version 2)*** |
| --- | --- |
| *Introduction section* | |
| *Covers questions relevant to recipients of JRA OT training:*  *Example question: Can you briefly walk me through the type of training you received on the use of the JRA OT for characterizing the risk of biological threats at the national and/or sub-national level?* | *Covers questions relevant to the design of the JRA OT and the providers of the training*  *Example questions:*  *Can you briefly walk me through the type of training provided on the use of the JRA OT for characterizing the risk of biological threats at the national and/or sub-national level?*  *Based on your experiences with past trainings, in what ways do you think stakeholder would most benefit from future related cross-sectoral trainings?* |
| *Section on resource mobilization* | |
| *Example question: In your opinion, what actions are required to ensure a systematic allocation of resources for the implementation of cross-sectoral risk management and risk communication interventions following an EID risk characterization?* | *Covers questions on the work of partner organizations with governments on resource mobilization.*  *Example question: How do partner organizations work with the government on resource allocation and mobilization required to implement recommended interventions identified following the JRA process?* |

Once again, thank you for further considering our manuscript for publication in your journal. I hope you find the points addressed in the resubmitted version of the manuscript to your satisfaction. We look forward to hearing from you in this regard.

Sincerely,

Oluwayemisi Ajumobi
