# Supplementary material for: Improving risk analysis of environmentally driven zoonotic biological threats as a primary pandemic prevention approach: A case study of the Tripartite Joint Risk Assessment Operational Tool operationalization in Kenya
Source: PLOS Glob Public Health. 2026 Jul 1;6(7):e0006560. doi: 10.1371/journal.pgph.0006560 (PMC13322548; doi:10.1371/journal.pgph.0006560)
Supplement: Peer review history

# Peer review: Decision Letter 0

**DOI:** 10.1371/journal.pgph.0006560.r001

**Authors:** Syed Shahid Abbas

30 Apr 2025

PGPH-D-25-00514

Improving risk analysis of environmentally driven zoonotic biological threats as a primary pandemic prevention approach: A case study of the Tripartite Joint Risk Assessment Operational Tool operationalization in Kenya

PLOS Global Public Health

Dear Dr. Ajumobi,

Thank you for submitting your manuscript to PLOS Global Public Health. After careful consideration, we feel that it has merit but does not fully meet PLOS Global Public Health’s publication criteria as it currently stands. Therefore, we invite you to submit a revised version of the manuscript that addresses the points raised during the review process.

Please refer to additional Editor comments (below) in addition to reviewer comments.

Please submit your revised manuscript by Jun 14 2025 11:59PM. If you will need more time than this to complete your revisions, please reply to this message or contact the journal office at globalpubhealth@plos.org. When you're ready to submit your revision, log on to https://www.editorialmanager.com/pgph/ and select the 'Submissions Needing Revision' folder to locate your manuscript file.

Please include the following items when submitting your revised manuscript:

- A rebuttal letter that responds to each point raised by the editor and reviewer(s). You should upload this letter as a separate file labeled 'Response to Reviewers'.
- A marked-up copy of your manuscript that highlights changes made to the original version. You should upload this as a separate file labeled 'Revised Manuscript with Track Changes'.
- An unmarked version of your revised paper without tracked changes. You should upload this as a separate file labeled 'Manuscript'.

Guidelines for resubmitting your figure files are available below the reviewer comments at the end of this letter.

We look forward to receiving your revised manuscript.

Kind regards,

Syed Shahid Abbas, MBBS, MPH, Ph.D.

Academic Editor

PLOS Global Public Health

Journal Requirements:

1. Please include a complete copy of PLOS’ questionnaire on inclusivity in global research in your revised manuscript. Our policy for research in this area aims to improve transparency in the reporting of research performed outside of researchers’ own country or community. The policy applies to researchers who have travelled to a different country to conduct research, research with Indigenous populations or their lands, and research on cultural artefacts. The questionnaire can also be requested at the journal’s discretion for any other submissions, even if these conditions are not met. Please find more information on the policy and a link to download a blank copy of the questionnaire here: https://journals.plos.org/globalpublichealth/s/best-practices-in-research-reporting. Please upload a completed version of your questionnaire as Supporting Information when you resubmit your manuscript.

2. Please provide separate figure files in .tif or .eps format.

For more information about figure files please see our guidelines: https://journals.plos.org/globalpublichealth/s/figures

https://journals.plos.org/globalpublichealth/s/figures#loc-file-requirements

Additional Editor Comments (if provided):

1- Please refer to comments from both the reviewers on the aims and design of your study, including the additional value from an IRA.

2- It is not clear to me how you understand environment health / hazards and also what might 'environment sector' mean in the context of your study site (Kenya) as well as more widely in other countries. It will be helpful if you could define these concepts and clarify your stance.

[Note: HTML markup is below. Please do not edit.]

Reviewers' comments:

Reviewer's Responses to Questions

**Comments to the Author**

1. Does this manuscript meet PLOS Global Public Health’s publication criteria? Is the manuscript technically sound, and do the data support the conclusions? The manuscript must describe methodologically and ethically rigorous research with conclusions that are appropriately drawn based on the data presented.

Reviewer #1: Yes

Reviewer #2: Yes

**********

2. Has the statistical analysis been performed appropriately and rigorously?

Reviewer #1: N/A

Reviewer #2: Yes

**********

3. Have the authors made all data underlying the findings in their manuscript fully available (please refer to the Data Availability Statement at the start of the manuscript PDF file)?

The PLOS Data policy requires authors to make all data underlying the findings described in their manuscript fully available without restriction, with rare exception. The data should be provided as part of the manuscript or its supporting information, or deposited to a public repository. For example, in addition to summary statistics, the data points behind means, medians and variance measures should be available. If there are restrictions on publicly sharing data—e.g. participant privacy or use of data from a third party—those must be specified.

Reviewer #1: Yes

Reviewer #2: Yes

**********

4. Is the manuscript presented in an intelligible fashion and written in standard English?

PLOS Global Public Health does not copyedit accepted manuscripts, so the language in submitted articles must be clear, correct, and unambiguous. Any typographical or grammatical errors should be corrected at revision, so please note any specific errors here.

Reviewer #1: Yes

Reviewer #2: No

**********

5. Review Comments to the Author

Please use the space provided to explain your answers to the questions above. You may also include additional comments for the author, including concerns about dual publication, research ethics, or publication ethics. (Please upload your review as an attachment if it exceeds 20,000 characters)

Reviewer #1: Overall:

This paper provides important information on application of the JRA tool from users in Kenya. The key themes that emerged were very interesting and operationally important, but none are excluded within the existing JRA process. The relationship of the proposed IRA and the existing JRA OT (as well as the other RA frameworks mentioned) is unclear, particularly the specifics of the proposed IRA and how the proposed IRA differs in operational substance from the JRA.

Similarly, it is unclear why improved application of the existing JRA tool by inclusion / integration of information and actors from the environment sector, rather than creating a new framework with a new name, would be required to account for e.g. environmental drivers and why the other aspects of the output triad could not fit in the existing JRA process.

The main added value seems to be a recommendation of ensuring the JRA risk management and communication outcomes are implemented in policy including environmental impact assessments, but no specific operational solution is provided. Would seem to be more useful to present the information as specific guidance to improve scope and sustainability of outcomes for those using the JRA and practical ways of working outcomes into policy.

Specific comments

Summary: The environmental aspects highlighted were not included specifically as essential component outcomes of the interviews. Need to link/align results with analysis

P4 ‘The JRA OT is one of three operational tools … to qualitatively assess risks posed by zoonotic disease hazards at the human-animal-environment interfaces, and at the national and sub-national levels.’ – there are not three RA OT tools, but three tools overall.

Describe any efforts at improving the gender balance as well as the balance across sectors and disciplines and specifically how that could have impacted the results.

Quality of fig 1 could be improved, including what aspects are included in the JRA and the other RA frameworks mentioned.

Reviewer #2: Congratulations to the authors of this study! In my opinion, this study aimed to evaluate the usefulness of the Joint Risk Assessment (JRA) Operational Tool, focusing on conducting integrated risk assessments of infectious disease risk caused by environmental changes. I find the topic highly relevant because, to harvest the benefits of the One Health approach, all stakeholders must actively participate in and contribute to One Health initiatives. In my experience, bringing the human and animal health sectors together has been a challenge, but not as challenging as getting the environmental sector on board. The challenge is reflected in the late addition of UNEP to the institutions supporting the JRA tool. I applaud the authors' efforts to bring the environmental perspective into the debate. I believe the timing is opportune because, as the authors mention, the increase in extreme weather events may increase the threat of emerging or re-emerging infectious diseases. In my opinion, the impact of this study is the development of a new integrated risk analysis (IRA) framework that better incorporates the environmental sector, in addition to addressing other areas that the study identifies as areas for improvement.

Major issues

1. I suggest that the authors review the aim of their study. In my opinion, the aim is to evaluate the usefulness of the JRA-OT or the usefulness of the current IRA framework. This is because:

a. The participants were required to be acquainted with the JRA-OT, and many of the questions were about the JRA-OT, not other frameworks.

b. The abstract states that the aim is to “obtain the perspectives of stakeholders on the development…” However, the participants were not asked for their perspectives on a new proposed IRA framework, but rather their experiences with the current framework.

c. The four major themes that emerged from the study are not limited to risk analysis for infectious diseases driven by environmental changes. The improved IRA will not only address the inclusion of the environmental sector, but also these four themes.

Minor issues

1. Abstract: The abbreviation “JRA” is not defined in the abstract. Add what JRA stands for in the abstract.

2. Introduction: To the sentence “Hence, in addition to priority endemic zoonotic diseases (such as anthrax, brucellosis, bovine tuberculosis, and rabies)” provide a reference. Who established these diseases as priorities?

3. Introduction: Are the pathogens of focus, CCHFV, MARV, and MERS-CoV, environmentally driven? According to the introduction, only MARV is environmentally driven.

4. Introduction: Describe what the studies 13-15 claimed. Explain what the evidence or lack of evidence exists to call for “a better understanding and synthesis of probable environmentally driven disease transmission risk pathways”.

5. Methods: In the description of the eligibility criteria, clarify whether all criteria were required (AND) or only one (OR).

6. Methods: What were the categories (and the weight of each category) planned for the stratified sampling?

7. Methods: I suggest moving the number of declinations to participate to the results section. Describe the characteristics of those who decline. Were they appointed to the government or the quadripartite institutions?

8. Methods: Some interviews were conducted at work. How was privacy safeguarded? Is it possible that participants felt obliged to respond in a certain way to please the view of their institution?

9. Methods: I suggest not referencing a framework located in the protocol unless the protocol can be accessed.

10. Methods: Clarify how INFORM GRI and Health EDRM were used.

11. Methods: The description of Table 1 in the methods may not correspond to Table 1 in Results.

12. Results: Describe the 14 participants included in the validation workshop. Comment about the representativeness of the sample to the interviewed population.

13. Discussion: Please clarify how this study aligns with “data-driven risk analysis-based strategies”. The JAR-OT is a qualitative tool that could rely on expert opinion.

14. Discussion: Consider comparing the usefulness of INFORM GRI, Health EDRM, and JAR, focusing on the environmental component of OH.

15. Discussion: What policies are currently in place in Kenya? In the discussion, the authors indicate the “need for the development and implementation of OH policies.” What should be included in these policies?

16. Supplement: What are the main differences between versions 1 and 2 of the semi-structured interview guides?

**********

6. PLOS authors have the option to publish the peer review history of their article (what does this mean?). If published, this will include your full peer review and any attached files.

**Do you want your identity to be public for this peer review?**   If you choose “no”, your identity will remain anonymous but your review may still be made public.

For information about this choice, including consent withdrawal, please see our Privacy Policy.

Reviewer #1: **Yes:**   Elizabeth Mumford

Reviewer #2: No

**********

[NOTE: If reviewer comments were submitted as an attachment file, they will be attached to this email and accessible via the submission site. Please log into your account, locate the manuscript record, and check for the action link "View Attachments". If this link does not appear, there are no attachment files.]

While revising your submission, please upload your figure files to the Preflight Analysis and Conversion Engine (PACE) digital diagnostic tool, https://pacev2.apexcovantage.com/. PACE helps ensure that figures meet PLOS requirements. To use PACE, you must first register as a user. Registration is free. Then, login and navigate to the UPLOAD tab, where you will find detailed instructions on how to use the tool. If you encounter any issues or have any questions when using PACE, please email PLOS at figures@plos.org. Please note that Supporting Information files do not need this step.

# Author comment: Author response to Decision Letter 1

**DOI:** 10.1371/journal.pgph.0006560.r002

6 Nov 2025

Attachment Submitted filename: Response to reveiwers.docx

# Peer review: Decision Letter 1

**DOI:** 10.1371/journal.pgph.0006560.r003

**Authors:** Syed Shahid Abbas

1 Dec 2025

PGPH-D-25-00514R1

Improving risk analysis of environmentally driven zoonotic biological threats as a primary pandemic prevention approach: A case study of the Tripartite Joint Risk Assessment Operational Tool operationalization in Kenya

PLOS Global Public Health

Dear Dr. Ajumobi,

Thank you for submitting your manuscript to PLOS Global Public Health. After careful consideration, we feel that it has merit but does not fully meet PLOS Global Public Health’s publication criteria as it currently stands. Therefore, we invite you to submit a revised version of the manuscript that addresses the points raised during the review process.

Please submit your revised manuscript by Jan 15 2026 11:59PM. If you will need more time than this to complete your revisions, please reply to this message or contact the journal office at globalpubhealth@plos.org. When you're ready to submit your revision, log on to https://www.editorialmanager.com/pgph/ and select the 'Submissions Needing Revision' folder to locate your manuscript file.

Please include the following items when submitting your revised manuscript:

- A rebuttal letter that responds to each point raised by the editor and reviewer(s). You should upload this letter as a separate file labeled 'Response to Reviewers'.
- A marked-up copy of your manuscript that highlights changes made to the original version. You should upload this as a separate file labeled 'Revised Manuscript with Track Changes'.
- An unmarked version of your revised paper without tracked changes. You should upload this as a separate file labeled 'Manuscript'.

Guidelines for resubmitting your figure files are available below the reviewer comments at the end of this letter.

We look forward to receiving your revised manuscript.

Kind regards,

Syed Shahid Abbas, MBBS, MPH, Ph.D.

Academic Editor

PLOS Global Public Health

Journal Requirements:

1. Please include a complete copy of PLOS’ questionnaire on inclusivity in global research in your revised manuscript. Our policy for research in this area aims to improve transparency in the reporting of research performed outside of researchers’ own country or community. The policy applies to researchers who have travelled to a different country to conduct research, research with Indigenous populations or their lands, and research on cultural artefacts. The questionnaire can also be requested at the journal’s discretion for any other submissions, even if these conditions are not met. Please find more information on the policy and a link to download a blank copy of the questionnaire here: https://journals.plos.org/globalpublichealth/s/best-practices-in-research-reporting. Please upload a completed version of your questionnaire as Supporting Information when you resubmit your manuscript.

If the reviewer comments include a recommendation to cite specific previously published works, please review and evaluate these publications to determine whether they are relevant and should be cited. There is no requirement to cite these works unless the editor has indicated otherwise.

Additional Editor Comments (if provided):

[Note: HTML markup is below. Please do not edit.]

Reviewers' comments:

Reviewer's Responses to Questions

**Comments to the Author**

1. If the authors have adequately addressed your comments raised in a previous round of review and you feel that this manuscript is now acceptable for publication, you may indicate that here to bypass the “Comments to the Author” section, enter your conflict of interest statement in the “Confidential to Editor” section, and submit your "Accept" recommendation.

Reviewer #1: (No Response)

Reviewer #2: All comments have been addressed

**********

2. Does this manuscript meet PLOS Global Public Health’s publication criteria? Is the manuscript technically sound, and do the data support the conclusions? The manuscript must describe methodologically and ethically rigorous research with conclusions that are appropriately drawn based on the data presented.

Reviewer #1: Partly

Reviewer #2: Yes

**********

3. Has the statistical analysis been performed appropriately and rigorously?

Reviewer #1: N/A

Reviewer #2: N/A

**********

4. Have the authors made all data underlying the findings in their manuscript fully available (please refer to the Data Availability Statement at the start of the manuscript PDF file)?

The PLOS Data policy requires authors to make all data underlying the findings described in their manuscript fully available without restriction, with rare exception. The data should be provided as part of the manuscript or its supporting information, or deposited to a public repository. For example, in addition to summary statistics, the data points behind means, medians and variance measures should be available. If there are restrictions on publicly sharing data—e.g. participant privacy or use of data from a third party—those must be specified.

Reviewer #1: Yes

Reviewer #2: Yes

**********

5. Is the manuscript presented in an intelligible fashion and written in standard English?

PLOS Global Public Health does not copyedit accepted manuscripts, so the language in submitted articles must be clear, correct, and unambiguous. Any typographical or grammatical errors should be corrected at revision, so please note any specific errors here.

Reviewer #1: Yes

Reviewer #2: Yes

**********

6. Review Comments to the Author

Please use the space provided to explain your answers to the questions above. You may also include additional comments for the author, including concerns about dual publication, research ethics, or publication ethics. (Please upload your review as an attachment if it exceeds 20,000 characters)

Reviewer #1: Thank you for the explanations provided in your response to editors and reviewers comments. This helped to clarify the objectives and outcomes of the research and the timing/positionality of IRA and JRA enormously. However, with the greater understanding you offered in your responses, I am afraid that more serious concerns emerge emphasizing that the current presentation of your framework was not – and remains not - completely clear.

1. The abstract refers to the IRA but gives no indication where this framework comes from and where it in in time in relation to this paper – does it already exist? Is this research informing its development? Are you presenting your framework here, as figure 1? If it is figure 1 (although it seems to only be a component of figure 1), the paper does not seem to actually present it and the data obtained in this study would anyway be insufficient for developing an entire framework.

2. Similarly, the cover letter suggests the newly developed IRA will be presented, and the paper refers to the development of such a a newly conceptualized integrated framework for risk analyses but the abstract states ‘Improving IRA processes… will…’ suggesting that IRA processes already exist, but also states that ‘Information obtained from the study was used to finalize the development of an integrated risk analysis (IRA) framework for pathogen/disease spillover, emergence/reemergence and spread (SES) risk analysis to inform cross-sectoral pandemic prevention decision making’ yet, again, the framework seems not to actually be presented/described in the paper and there is no mention of where that framework might be found. All this, including where the framework is, must be explained and clarified in the abstract and early in the paper.

3. The title should be changed to highlight that this is a qualitative study to learn mostly about integrating environment in RA, based partially on application of the JRA IN KENYA, not a case study or evaluation of the JRA tool itself (as per response to reviewer 2) and certainly not its operationalization. The cover letter also suggests Kenya will be used as a case study, which is truer than suggesting that the JRA will be used as one. The abstract – which I think describes the study quite accurately, only peripherally refers to the JRA.

4. Please correct WOOAH

5. Is the term conceptual framework referring to the IRA framework? Please clarify use of the terms.

6. Please comment/clarify in the document the extent of difference or overlap of i – iv concepts with basic principles of the JRA OT itself

Also, although you made some smaller changes and clarified several more complex points for the editor and reviewer in your responses, your comments did not translate into edits allowing better understanding for the reader. These, and a reflexive review of the above concerns, should allow development of a clearer presentation within the paper of these issues.

Reviewer #2: (No Response)

**********

7. PLOS authors have the option to publish the peer review history of their article (what does this mean?). If published, this will include your full peer review and any attached files.

**Do you want your identity to be public for this peer review?**   If you choose “no”, your identity will remain anonymous but your review may still be made public.

For information about this choice, including consent withdrawal, please see our Privacy Policy.

Reviewer #1: No

Reviewer #2: No

**********

[NOTE: If reviewer comments were submitted as an attachment file, they will be attached to this email and accessible via the submission site. Please log into your account, locate the manuscript record, and check for the action link "View Attachments". If this link does not appear, there are no attachment files.]

-->Figure Resubmissions:

While revising your submission, we strongly recommend that you use PLOS’s NAAS tool (https://ngplosjournals.pagemajik.ai/artanalysis) to test your figure files. NAAS can convert your figure files to the TIFF file type and meet basic requirements (such as print size, resolution), or provide you with a report on issues that do not meet our requirements and that NAAS cannot fix.

After uploading your figures to PLOS’s NAAS tool - https://ngplosjournals.pagemajik.ai/artanalysis, NAAS will process the files provided and display the results in the "Uploaded Files" section of the page as the processing is complete. If the uploaded figures meet our requirements (or NAAS is able to fix the files to meet our requirements), the figure will be marked as "fixed" above. If NAAS is unable to fix the files, a red "failed" label will appear above. When NAAS has confirmed that the figure files meet our requirements, please download the file via the download option, and include these NAAS processed figure files when submitting your revised manuscript.

# Author comment: Author response to Decision Letter 2

**DOI:** 10.1371/journal.pgph.0006560.r004

11 Apr 2026

Attachment Submitted filename: Updated response to peer reviewer_042026.pdf

# Peer review: Decision Letter 2

**DOI:** 10.1371/journal.pgph.0006560.r005

**Authors:** Syed Shahid Abbas

13 May 2026

Improving risk analysis of environmentally driven zoonotic biological threats as a primary pandemic prevention approach: A case study of the Tripartite Joint Risk Assessment Operational Tool operationalization in Kenya

PGPH-D-25-00514R2

Dear Dr Ajumobi,

We are pleased to inform you that your manuscript 'Improving risk analysis of environmentally driven zoonotic biological threats as a primary pandemic prevention approach: A case study of the Tripartite Joint Risk Assessment Operational Tool operationalization in Kenya' has been provisionally accepted for publication in PLOS Global Public Health.

Before your manuscript can be formally accepted you will need to complete some formatting changes, which you will receive in a follow up email. A member of our team will be in touch with a set of requests.

Please note that your manuscript will not be scheduled for publication until you have made the required changes, so a swift response is appreciated.

IMPORTANT: The editorial review process is now complete. PLOS will only permit corrections to spelling, formatting or significant scientific errors from this point onwards. Requests for major changes, or any which affect the scientific understanding of your work, will cause delays to the publication date of your manuscript.

If your institution or institutions have a press office, please notify them about your upcoming paper to help maximize its impact. If they'll be preparing press materials, please inform our press team as soon as possible -- no later than 48 hours after receiving the formal acceptance. Your manuscript will remain under strict press embargo until 2 pm Eastern Time on the date of publication. For more information, please contact globalpubhealth@plos.org.

Thank you again for supporting Open Access publishing; we are looking forward to publishing your work in PLOS Global Public Health.

Best regards,

Syed Shahid Abbas, MBBS, MPH, Ph.D.

Academic Editor

PLOS Global Public Health

***********************************************************

Reviewer Comments (if any, and for reference):

Reviewer's Responses to Questions

**Comments to the Author**

1. If the authors have adequately addressed your comments raised in a previous round of review and you feel that this manuscript is now acceptable for publication, you may indicate that here to bypass the “Comments to the Author” section, enter your conflict of interest statement in the “Confidential to Editor” section, and submit your "Accept" recommendation.

Reviewer #1: All comments have been addressed

**********

2. Does this manuscript meet PLOS Global Public Health’s publication criteria? Is the manuscript technically sound, and do the data support the conclusions? The manuscript must describe methodologically and ethically rigorous research with conclusions that are appropriately drawn based on the data presented.

Reviewer #1: Yes

**********

3. Has the statistical analysis been performed appropriately and rigorously?

Reviewer #1: N/A

**********

4. Have the authors made all data underlying the findings in their manuscript fully available (please refer to the Data Availability Statement at the start of the manuscript PDF file)?

The PLOS Data policy requires authors to make all data underlying the findings described in their manuscript fully available without restriction, with rare exception. The data should be provided as part of the manuscript or its supporting information, or deposited to a public repository. For example, in addition to summary statistics, the data points behind means, medians and variance measures should be available. If there are restrictions on publicly sharing data—e.g. participant privacy or use of data from a third party—those must be specified.

Reviewer #1: Yes

**********

5. Is the manuscript presented in an intelligible fashion and written in standard English?

PLOS Global Public Health does not copyedit accepted manuscripts, so the language in submitted articles must be clear, correct, and unambiguous. Any typographical or grammatical errors should be corrected at revision, so please note any specific errors here.

Reviewer #1: Yes

**********

6. Review Comments to the Author

Please use the space provided to explain your answers to the questions above. You may also include additional comments for the author, including concerns about dual publication, research ethics, or publication ethics. (Please upload your review as an attachment if it exceeds 20,000 characters)

Reviewer #1: N/a

**********

7. PLOS authors have the option to publish the peer review history of their article (what does this mean?). If published, this will include your full peer review and any attached files.

**Do you want your identity to be public for this peer review?**   If you choose “no”, your identity will remain anonymous but your review may still be made public.

For information about this choice, including consent withdrawal, please see our Privacy Policy.

Reviewer #1: **Yes:**   Elizabeth Mumford

**********
